# Hypoxia traits imprinted in otolith δ^13^C from individual to global scales

**DOI:** 10.1038/s41598-024-82518-0

**Published:** 2025-01-02

**Authors:** Evan M. Howard, Curtis A. Deutsch

**Affiliations:** 1 Department of Geosciences, Princeton University, Princeton, NJ 08540 USA; 2 Cooperative Institute for Climate, Ocean, and Ecosystem Studies, University of Washington, Seattle, WA 98195 USA; 3 High Meadows Environmental Institute, Princeton University, Princeton, NJ 08540 USA

**Keywords:** Marine biology, Metabolism

## Abstract

Hypoxia tolerance and its variation with temperature, activity, and body mass, are critical ecophysiological traits through which climate impacts marine ectotherms. To date, experimental determination of these traits is limited to a small subset of modern species. We leverage the close coupling of carbon and oxygen in animal metabolism to mechanistically relate these traits to the carbon isotopes in fish otoliths (δ^13^C_oto_). The model reproduces the major empirical patterns in δ^13^C_oto_ at individual to global scales. The weak dependence on body size and strong, non-linear, dependence on temperature reflect the same balance between metabolism and ventilatory gas exchange that underlies organisms’ hypoxia tolerance. The global relationship between temperature and δ^13^C_oto_ records both the fractionation by aragonite precipitation and the variation in hypoxia traits across ocean biomes. Because hypoxia tolerance is imprinted on both otolith geochemistry and species biogeography, the model allows the aerobic limits of species geographic ranges to be predicted from fish δ^13^C_oto_. This physiologically grounded model provides a foundation for the use of otolith chemistry to reconstruct modern spatial patterns and paleoceanographic changes in key traits that shape aerobic habitat of aquatic species.

## Introduction

The tolerance of marine species for low O_2_ and the changes in that tolerance with temperature, body size, and activity level have emerged as primary traits shaping marine ecosystems and their response to climate change. The physiological traits that quantify hypoxia tolerance and its sensitivities (hereafter ‘hypoxia traits’, see Table 1), which differ strongly across taxa, are traditionally derived from laboratory respirometry^1–3^. They predict key oceanic patterns of biogeography^4,5^ and biodiversity^6,7^, the response of body size to temperature^8–10^, the loss of habitat in historical observations^11–14^, and species extinction in the geologic record^15^. Direct determination of these hypoxia traits is limited to a small fraction of marine diversity, and robust estimation is hampered by strong variation among individuals and thermal non-linearities^16^. Biogeochemical signatures of hypoxia traits, if preserved and quantitatively retrievable from natural specimens, could shed new light on energetic flows and constraints on marine ecosystems in the modern ocean and in past climates.

**Table 1:** Glossary of parameters.

Parameter	Units	Definition
Isotopic and chemical variables		
δ^13^C (_oto_, _int_, _met_, _w_)	‰	δ = ([^13^C/^12^C]_measured_ / [^13^C/^12^C]_standard_ – 1) **·** 1000; otolith aragonite (CaCO_3_), internal fluid (CO_2(aq)_), metabolic carbon (DIC), and seawater (CO_2(aq)_)
Δ^13^C_A_(T)	‰	Temperature-dependent isotopic effect of equilibrium fractionation between CO_2(aq)_, and aragonite
P^C^ (_int_, _met_, _w_)	Atm	Partial pressure of CO_2_ of internal fluids, metabolic excess compared to seawater, and seawater
P^O^ (_met_, _w_)	Atm	Partial pressure of O_2_ of metabolic deficit compared to seawater, and seawater
Q(T)	Non-dimensional	Temperature-dependent stoichiometric coefficient
Hypoxia traits		
***V***_***h***_	Atm	Hypoxia vulnerability at a reference temperature and mass
***ε***	Non-dimensional	Allometric scaling exponent of supply to demand
***E***_***o***_	eV	Temperature scaling exponent of demand to supply
***SMS***	Non-dimensional	Sustained metabolic scope, the ratio of sustained to resting metabolic rates; the equivalent biogeographically derived value is termed ***Φ***_***c***_

Otoliths—ear stones—record the life history of fish. The carbon stable isotope ratio of otoliths (δ^13^C_oto_) reflects contributions of both organic carbon from the diet and dissolved inorganic carbon (DIC) from the surrounding water^17,18^. Recent work illustrates a link between otolith carbon isotopic composition and the sustained metabolic rates of individual fish^19–24^. However, interspecific relationships between δ^13^C_oto_ and key determinants of metabolic rate, including temperature and body size, are both weaker than predicted by scaling of metabolic rates alone^19^. Such discrepancies may in part reflect the fact that metabolic scaling is highly variable among species^25^. A more fundamental reason is that the pool of metabolic carbon that accumulates in body tissues depends not only on metabolism but equally on the rate at which it is ventilated to the environment, which also varies strongly with species, temperature, and body size. Because hypoxia tolerance is similarly governed by the ratio of the rates of metabolism to ventilation, the δ^13^C_oto_ of otoliths may instead reveal vital information about species critical physiological tolerances, in which metabolic rate is but one factor.

We derive a model to predict δ^13^C of otolith aragonite from fundamental physiological hypoxia traits and their variations across a range of environmental conditions, species, and body sizes. The model solves the carbon isotopic mass balance of a generic organism that exchanges carbon with its environment via food supply and gas exchange. Our approach differs from previous work in three ways. First, in contrast to diagnostic models, which use the otolith isotopic ratio to infer the fraction of metabolic carbon, our model predicts the internal pool of metabolic carbon from physiological traits, and these predictions are validated using direct measurements. Second, our model explicitly accounts for both the species-specific metabolic source and ventilatory loss of CO_2_, revealing that otolith ^13^C scales with the ratio of these physiological rates, which have been measured experimentally for many species. Third, we account for the speciation and fractionation of carbon pools necessary to represent gas exchange by pCO_2_ and the formation of otolith aragonite from DIC, respectively. The result is a set of model predictions that is consistent with multiple independent lines of evidence including hypoxia tolerance traits from respirometry, organismal pCO_2_ observations in blood, and otolith δ^13^C_oto_ across individuals, populations, and biomes.

The predictive nature of our model relies on the close coupling of the fluxes of oxygen and carbon in animal respiration; a dynamical representation of oxygen supply and demand and their variation with temperature and body size^16^; and independently measured physiological traits across marine ectotherms. A mechanistic model that combines these features shows that the δ^13^C of otoliths is intimately linked to—and can be predicted from—hypoxia tolerance measured across species, temperatures, and body sizes. This model framework allows otolith carbon isotope variations to be predicted at multiple scales, from the life history of individual organisms to variation among individuals and populations within a species, to biome scale differences across the global ocean. More importantly, we show that the model also works in reverse, predicting the hypoxic limits of species from their otolith carbon isotopes.

## Model: biogeochemical signature of physiological hypoxia traits

**Fig. 1:**
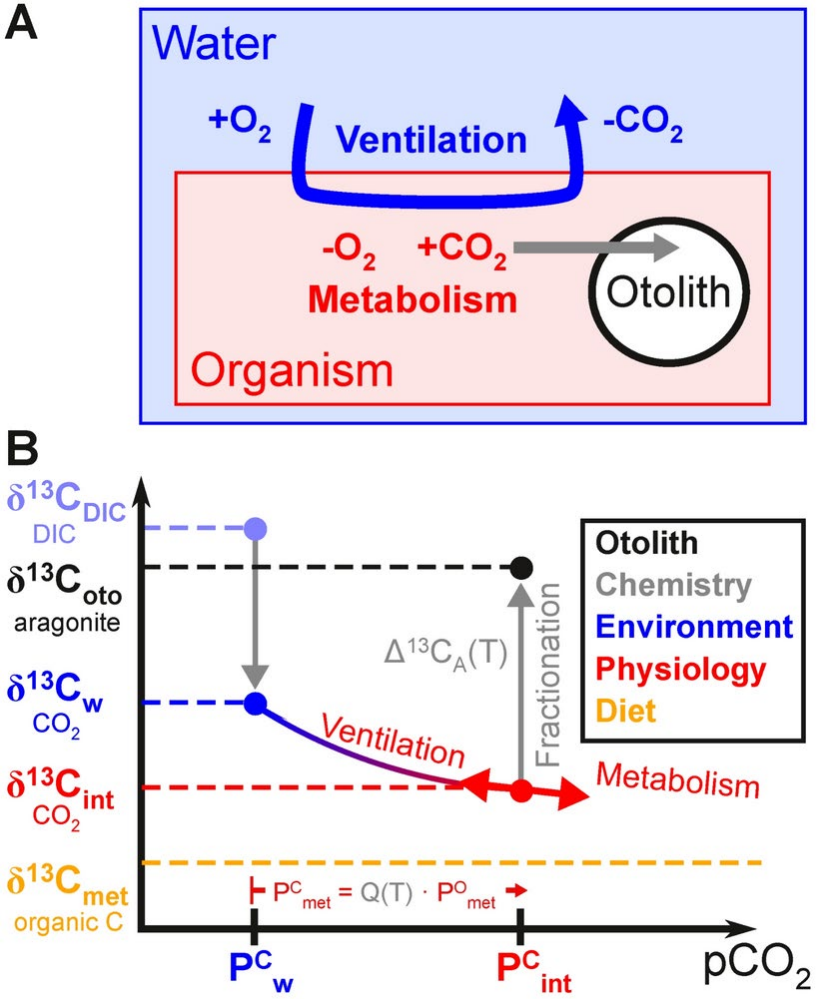
Schematic depiction of otolith carbon isotope model. (**A**) The physiological model includes carbon generated by metabolic processes and its ventilation to the environment, as CO_2_. Otolith aragonite is equilibrated with the internal carbon pool, which includes seawater and metabolic sources. (**B**) The isotopic composition of CO_2_ in internal fluids (δ^13^C_int_, as CO_2_; red) varies between that of metabolic carbon (δ^13^C_met_, derived from organic carbon in the diet; orange) and seawater carbon (δ^13^C_w_, as CO_2_; blue). The composition of δ^13^C_w_ is depleted by about 10 ‰ relative to the dissolved inorganic carbon is seawater (δ^13^C_DIC_, as DIC; light blue). The δ^13^C_int_ depends on the balance of physiological rates, and asymptotically approaches δ^13^C_met_ (curve) with increasing metabolism, which is opposed by carbon loss through the gills with ventilation. The net accumulation of metabolic carbon (P^C^_met_) in excess of seawater (P^C^_w_) is linearly related to the oxygen deficit relative to seawater (P^O^_met_) through the temperature-dependent stoichiometry of metabolism and ventilation (Q(T)); described by red arrow along x-axis). Otolith aragonite is precipitated from this internal carbon pool, with the temperature dependent isotope effect of equilibrium fractionation (Δ^13^C_A_(T); gray) increasing the resulting otolith δ^13^C_oto_ (aragonite; black) by about 13 ‰ relative to δ^13^C_int_. The legend describes the general sources of or controls on each form of carbon (shared colors) contributing to the otolith isotopic composition, including inorganic carbon speciation and fractionation (‘Chemistry,’ gray), environmental temperature and distributions of inorganic carbon (‘Environment,’ blue), the balance of physiological rates (‘Physiology,’ red), and the food sources and metabolic processing that generate metabolic carbon (‘Diet,’ orange).

We developed a predictive model of otolith carbon isotopes based on the joint carbon and oxygen exchanges between a generalized aquatic organism and its environment (Fig. 1A). The model combines physiological mechanisms of O_2_ supply and demand from the Metabolic Index framework^4,26^ with aqueous carbon chemistry and isotopic mass balance to predict otolith δ^13^C. A heuristic derivation is presented here, along with a glossary describing the key model parameters (Table 1), and a detailed derivation and justification are in the Supplemental Material (*SM*, Text S1).

The carbon isotope ratio of an otolith, δ^13^C_oto_, records the composition of internal fluids (δ^13^C_int_) plus the temperature-dependent fractionation imprinted by aragonite precipitation relative to CO_2_ (Δ^13^C_A_(T)), such that δ^13^C_oto _= δ^13^C_int_ + Δ^13^C_A_(T) (Fig. 1B, gray arrow). The δ^13^C_int_ originates from both metabolic (δ^13^C_met_) and seawater (δ^13^C_w_) sources, where the weighting of each pool is given by its CO_2_ partial pressure (P^C^), such that:1where the fractional term on the right side is the isotope ratio of internal CO_2_ (i.e. δ^13^C_int_). As respiratory carbon accumulates, δ^13^C_int_ falls below δ^13^C_w_ to asymptotically approach the δ^13^C_met_ derived from the diet (Fig. 1B, curved line). This expression is similar to previous diagnostic models except that the relevant isotopic ratios and weighting of pools are derived from a mechanistic mass balance in which loss of carbon via gas exchange is driven only by aqueous CO_2_ [blue] while the carbon source largely reflects the isotopic ratio of the organic carbon from the diet [red] (see *SM*, Text S2.4).

The amount of metabolic carbon in an organism rises with the rate of respiratory carbon production, and declines with the rate of ventilatory exchange of CO_2_ with the environment (Fig. 1B, red arrows). An analogous balance applies to the rates of organismal O_2_ supply and metabolic demand, yielding an internal O_2_ deficit relative to ambient seawater, $\text{P}_{\text{met}}^{\text{O}}$, that is proportional to the accumulated internal respiratory carbon: $\text{P}_{\text{met}}^{\text{C}}$ = Q(T) · $\text{P}_{\text{met}}^{\text{O}}$. The stoichiometric quotient, Q(T) accounts for the impact of distinct temperature-dependent solubilities and diffusivities governing the exchange rates of the two gases (*SM*, Text S2). The metabolic contribution to δ^13^C_oto_ can thus be diagnosed from hypoxia traits regulating the respiratory O_2_ deficit of organisms, $\text{P}_{\text{met}}^{\text{O}}$.

The O_2_ dynamics of aquatic respiration have been widely studied across diverse taxa, including fishes. As environmental O_2_ pressure declines, an organism’s internal O_2_ pressure eventually becomes too low to support metabolism; at this critical oxygen pressure threshold, the ambient $\text{P}_{\text{w}}^{\text{O}}$ approximates $\text{P}_{\text{met}}^{\text{O}}$ (*SM*, Text S2). The accumulated metabolic carbon is thus directly related to the lowest pO_2_ that can sustain an organism’s metabolic rate. This threshold pressure, which quantifies hypoxia vulnerability, is the inverse of hypoxia tolerance and is commonly measured in a resting state ($\text{P}_{\text{w}}^{\text{O}}$ is also commonly denoted $\text{pO}_{{2}}^{\text{crit}}$ or P_crit_)^1^. Hypoxia vulnerability is observed to vary with body size (B; normalized to a reference mass) and temperature (T) according to: $\text{P}_{\text{met}}^{\text{O}}$ = ***V***_***h***_ · B^−***ε***^ · *R*(***E***_***o***_,T), where ***V***_***h***_ applies at a reference temperature and body mass, *R* is the exponential ‘Arrhenius’ function of temperature (*SM*, Text S1) and the species-specific hypoxia traits ***ε*** and ***E***_***o***_ quantify the allometric and thermal sensitivity of hypoxia vulnerability^26^. An organism’s hypoxia vulnerability increases with T when ***E***_***o***_ > 0 (‘colder is better’) and increases with body size when ***ε*** < 0 (‘smaller is better’), as found in most species. Active organisms have greater oxygen demand, such that $\text{P}_{\text{met}}^{\text{O}}$ is increased by the ratio of sustained to resting metabolic demand, termed sustained metabolic scope (***SMS***)^27^. This final hypoxia trait depends on ecological context as well as physiology, because it includes energetically costly activities such as pursuing prey, avoiding predators, migrating, and reproducing.

Combining the preceding relationships, we arrive at a predictive otolith carbon isotope equation (see *SM*, Text S1):

2 $${\updelta }^{13}{\text{C}}_{\text{oto}}=\frac{{\updelta }^{13}{\text{C}}_{\text{met}}\left[\text{Q}\left(\text{T}\right) \cdot \boldsymbol{ }{{\varvec{V}}}_{{\varvec{h}}} \cdot \boldsymbol{ }{\text{B}}^{{-\varvec{\varepsilon}}} \cdot R\left({{\varvec{E}}}_{{\varvec{o}}},\text{T}\right) \cdot {\varvec{S}}{\varvec{M}}{\varvec{S}}\right] + {\updelta }^{13}{\text{C}}_{\text{w}}{\cdot \text{ P}}_{\text{w}}^{\text{C}}}{{ }_{ }\left[\text{Q}\left(\text{T}\right) \cdot \boldsymbol{ }{{\varvec{V}}}_{{\varvec{h}}} \cdot {\text{B}}^{{-\varvec{\varepsilon}}} \cdot R{({\varvec{E}}}_{{\varvec{o}}},\text{T}) \cdot {\varvec{S}}{\varvec{M}}{\varvec{S}}\right] + {\text{P}}_{\text{w}}^{\text{C}}}+{\Delta }^{13}{\text{C}}_{\text{A}}(\text{T})$$

The bracketed term is the metabolic carbon ($\text{P}_{\text{met}}^{\text{C}}$) and includes all the species-specific hypoxia traits (***bold italic font***), while the remaining terms are environmental and chemical parameters.

The model (Eq. 2) thus allows δ^13^C_oto_ to be predicted, rather than diagnosed, from environmental conditions and species hypoxia traits. The physiological hypoxia traits (***V***_***h***_, ***E***_***o***_, and ***ε***) have been compiled based on respirometry experiments for a wide range of marine species^4,8^ (*SM*, Fig. S1, Text S2). The values of environmental and diet parameters, which vary over space and time according to the distribution and speciation of carbon in the ocean ($\text{P}_{\text{w}}^{\text{C}}$, δ^13^C_w_), ocean temperature, and trophic level within the marine food web (δ^13^C_met_), are evaluated using both observational and climatological data.

## Trait-based predictions across scales

We tested key assumptions and predictions of the hypoxia trait-based otolith model against observations at multiple scales. First, we test the relationship between metabolic excess of CO_2_ and deficit of O_2_ (Eq. 2) by comparing measurements of internal pCO_2_ against external hypoxia thresholds measured by respirometry. Second, we test the long-term imprinting of hypoxia vulnerability on the otolith by comparing observed δ^13^C_oto_ to predictions based on species’ hypoxia traits at three scales: Intraspecific variability (differences between individuals across populations of the same species), life-history variability (time-resolved fluctuations within individual organisms), and global variability (biome-scale patterns across the global ocean).

### Hypoxia vulnerability versus respiratory CO_2_

**Fig. 2:**
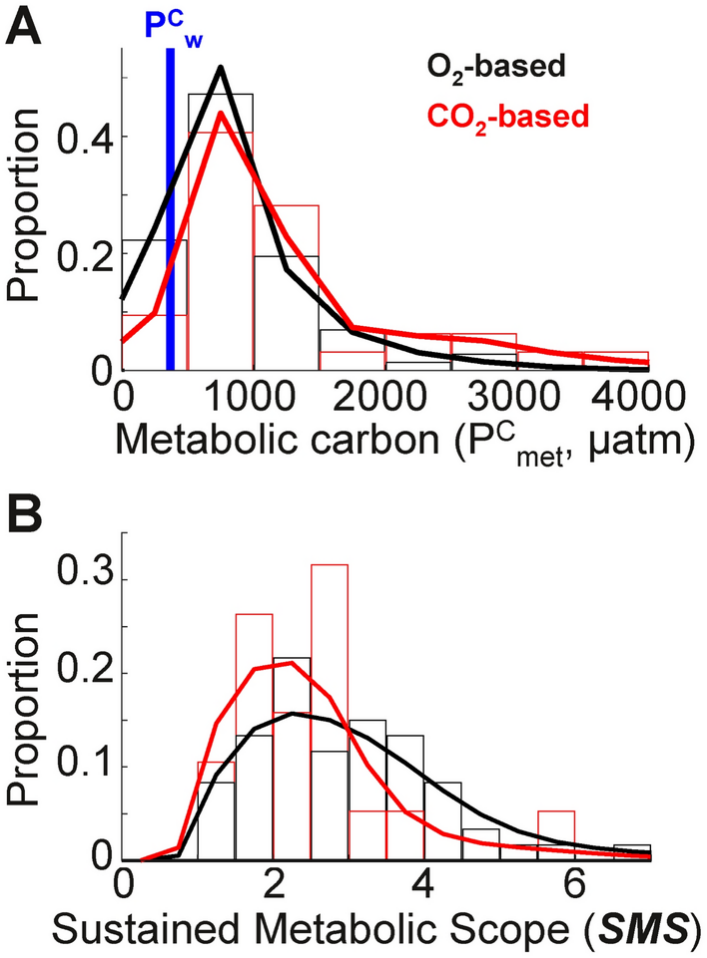
Internal CO_2_ from fish blood measurements compared to oxygen-based estimates. (**A**) Metabolic carbon distributions calculated from oxygen respirometry (‘O_2_-based’ $\text{P}_{\text{met}}^{\text{C}}$, using Q(T); black histogram and fitted probability distribution, n = 72) and from direct measurements of carbon in internal fluids (‘CO_2_-based’ $\text{P}_{\text{met}}^{\text{C}}$, calculated from $\text{P}_{\text{int}}^{\text{C}}$ or DIC; red histogram and fitted probability distribution, n = 32). Only observations for resting or low-activity animals are plotted, and normalized to a shared temperature (15 °C) and environmental carbon ($\text{P}_{\text{w}}^{\text{C}}$ = 370 µatm; blue line) as discussed in the *SM*, Text S3. Metabolic carbon exceeds environmental carbon in every experiment in which both internal and environmental carbon were directly measured (*SM*, Table S1). (**B**) Distributions of ***SMS*** calculated from oxygen biogeography (black, n = 72) and from direct measurements of carbon in internal fluids in paired experiments at rest and during exercise (n = 19).

We first test the relationship between hypoxia vulnerability and the CO_2_ in internal fluids, by predicting the interspecific distribution of $\text{P}_{\text{met}}^{\text{C}}$ using a database of measured critical O_2_ pressures for marine species^4^. The combination of hypoxia vulnerability (***V***_***h***_) and stoichiometric scaling, Q(T), yield values of $\text{P}_{\text{met}}^{\text{C}}$ roughly 1–4 times the pCO_2_ of ambient seawater, implying that metabolically-derived carbon often dominates the internal CO_2_ pool. To evaluate the consistency of model predicted $\text{P}_{\text{met}}^{\text{C}}$, we compiled data on the pCO_2_ in the fluids of fish (*SM*, Table S1, Fig. S2, and Text S3). While differences in taxonomic groups and experimental conditions make such comparisons approximate, the interspecific distribution of $\text{P}_{\text{met}}^{\text{C}}$ from direct carbon measurements in resting organisms is consistent with the model prediction based on hypoxia traits (Fig. 2A; Table 2).

**Table 2:** Test results.

Result	Test	Null hypothesis	*p*-value^a^	Test statistic	Obs. #
$\text{P}_{\text{met}}^{\text{C}}$ from O_2_ and CO_2_ have similar distributions	Two sample Kolmogorov–Smirnov test	$\text{P}_{\text{met}}^{\text{C}}$ estimates drawn from same distribution	0.10	D_n,m_ = 0.25	n = 72, m = 32
***SMS*** from O_2_ and CO_2_ have similar distributions	Two sample Kolmogorov–Smirnov test	***SMS*** estimates drawn from same distribution	0.07	D_n,m_ = 0.33	n = 72, m = 19
Reared Atlantic cod Δ^13^C_oto_ better fit by temperature-dependence ***E***_***o***_ than by *E*_*d*_	Akaike information criterion weight ratio	Model with *E*_*d*_ (metabolism only) better fits data	2 × 10^–10^	P_AICc,***Eo***_ = 1.00^b^	n = 88
Atlantic cod Δ^13^C_oto_ uncorrelated to log(***B***)	Linear correlations, least absolute residual weights^c^	No empirical mass dependence of Δ^13^C_oto_	0.11	F = 2.53	n = 147
Atlantic cod otolith-derived $\text{P}_{\text{met}}^{\text{O}}$ has little allometric dependence		No allometric dependence of $\text{P}_{\text{met}}^{\text{O}}$ (***ε*** = 0)	0.17	F = 1.94	n = 144^d^
***SMS*** from reared and wild Atlantic cod have different distributions	Two sample Kolmogorov–Smirnov test	***SMS*** estimates drawn from same distribution	4 × 10^–21^	D_n,m_ = 0.85	n = 98, m = 46
Otolith hypoxia thresholds match O_2_ approaches	Paired-sample t-test^e^	No systematic difference in estimates	0.23	t = 1.28	n = 12^f^
Otolith hypoxia thresholds correlated to O_2_ approaches	Linear correlation, linear least squares	No correlation between methods	7 × 10^–5^	F = 41.52	n = 12

^a^All tests assume α = 0.05 when determining significance.

^b^P_AICc,***Eo***_ is the normalized probability ratio of the weights (*w*) of the Akaike information criterion corrected for finite sampling (AICc); P_AICc,***Eo***_ = *w*_***Eo***_/( *w*_***Eo***_ + *w*_Ed_) = 1, while P_AICc,Ed_ = 2 × 10^–10^; an empirical likelihood function was used. The associated p-value was calculated using a χ^2^ test based on the observed difference between AICc for each model.

^c^Least absolute residual weights are less sensitive than least squared weights to heteroscedasticity introduced by the variations in sampling intensity across the range of body sizes.

^d^Temperature-normalized $\text{P}_{\text{met}}^{\text{O}}$ solved from measured isotopic values using literature hypoxia traits for Atlantic cod (including resting ***SMS*** = 1 for reared and ***SMS*** = ***Φ***_***c***_ = 2.4 for wild animals). Three wild samples with unconstrained estimates were excluded (*SM*, Text S6).

^e^Estimates and paired differences were confirmed to be approximately normally distributed.

^f^Individual samples resulting in unconstrained estimates (*SM*, Text S6) were excluded prior to calculating the species-mean value. Multiple Atlantic cod datasets were merged into a single wild and single reared set, and two marlin species had no well-constrained estimates. Errors were confirmed to be approximately normally distributed, with regression fit $\text{P}_{\text{w,biogeography}}^{\text{O}}$ = 0.007(0.022 to 0.037, 95%CI) + 0.988(0.679 to 1.298, 95% CI)·$\text{P}_{\text{w,otolith}}^{\text{O}}$, R^2^ = 0.84.

Blood carbon measured in organisms that are exercised compared to those at rest (*SM*, Table S1) yield an estimate of the ratio of active to resting metabolic rates (***SMS***). This ratio has previously been estimated directly from the ratio of active to resting rates of respiration, and indirectly from active to resting thresholds of hypoxia tolerance at a species level (termed ***Φ***_***c***_)^28^. The distribution of ***SMS*** from blood carbon agrees well with that inferred from O_2_-based metrics (Fig. 2B; Table 2). The theoretical and observed coupling of $\text{P}_{\text{met}}^{\text{C}}$ and $\text{P}_{\text{met}}^{\text{O}}$ supports the use of respirometry-derived hypoxia traits to predict internal metabolic carbon in both resting and active organisms.

The large observed contribution of $\text{P}_{\text{met}}^{\text{C}}$ to internal carbon contradicts the prevailing interpretation from prior isotopic analyses, that the carbon in otoliths comes primarily from seawater (e.g.,^17,29^; *SM*, Text S1.2). The discrepancy stems from the fact that the exchange of carbon with seawater imparts the isotopic ratio of CO_2_, which is significantly depleted in ^13^C relative to DIC used in previous studies. The higher proportion of metabolic carbon in our model thus reflects the true isotopic signal of ventilatory exchange, as well as being consistent with the blood data (Fig. 1B).

### Intraspecific variability

To test model predictions for intraspecific patterns of δ^13^C_oto_, we used Atlantic cod (*Gadus morhua*), a species for which both hypoxia tolerances and isotopic compositions have been measured across a wide range of temperatures (2–14 °C), body sizes (0.8–6700 g), and activity levels in wild and reared fish^19,30–32^. Long-term otolith isotope composition can thus be predicted using physiological hypoxia traits (***V***_***h***_, ***E***_***o***_, and **ε**) derived from short-term respirometry^4^. The hypoxia traits of Atlantic cod are also close to the average of all measured species, making modeled δ^13^C_oto_ patterns a useful reference point for otoliths of other species.

**Fig. 3:**
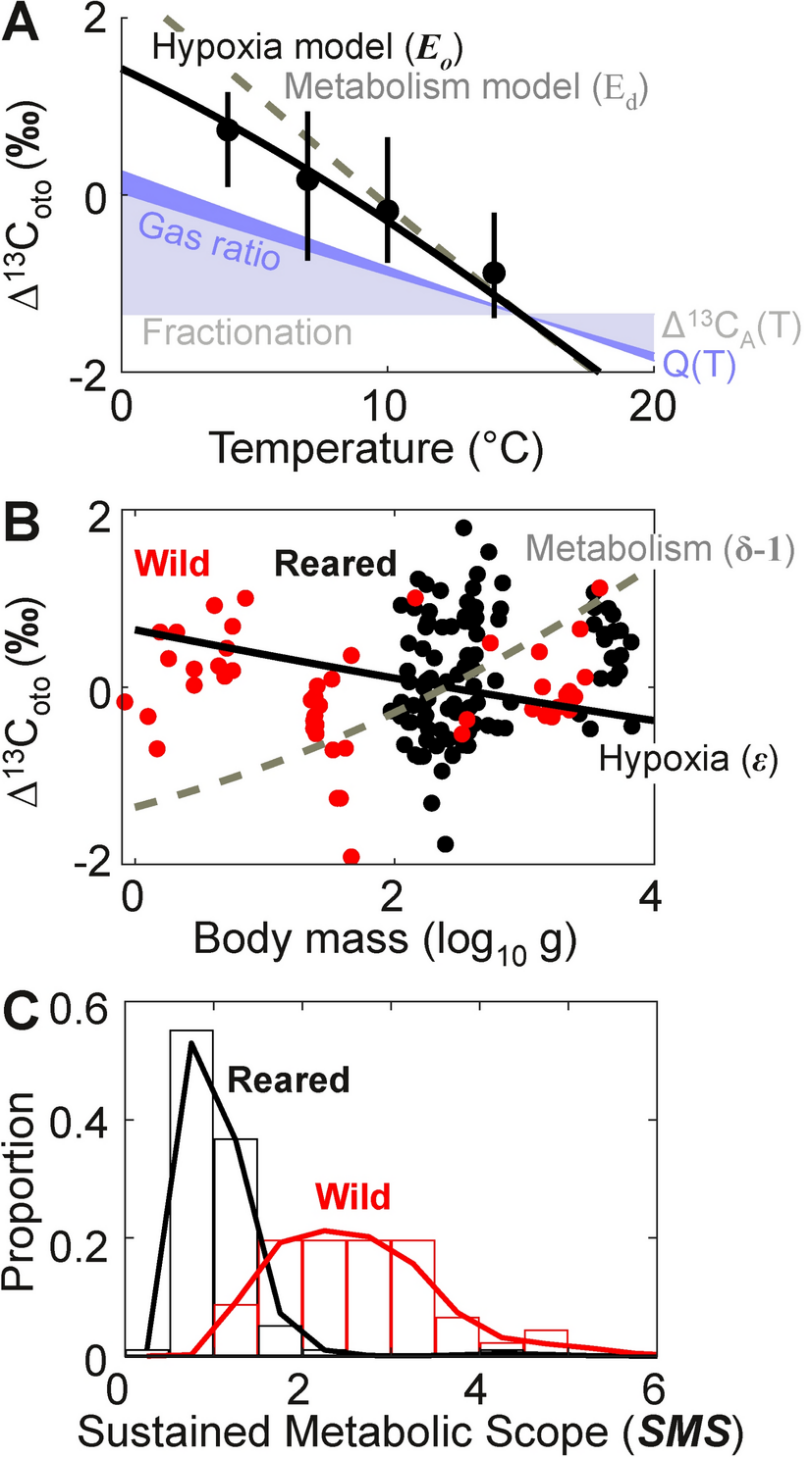
Comparison of δ^13^C_oto_ observations and model predictions for Atlantic cod. (**A**) The otolith δ^13^C versus temperature, plotted as a difference relative to the hypoxia model value at 8.5 °C (denoted Δ^13^C_oto_). Observational data is from reared cod (black dots are mean, bars are full range) with similar mass and diets. The shaded wedges represent the cumulative contribution of modeled chemical terms contributing to the temperature-dependence of Δ^13^C_oto_ (relative to the 15 °C reference temperature for hypoxia traits and intercept for model comparison). Chemical terms include the isotope fractionation effect (Δ^13^C_A_(T); light gray-blue) and, stacked above, the stoichiometric quotient (Q(T); light blue). The full physiological model (‘Hypoxia model,’ solid black curve) includes both those effects as well as the additional net temperature sensitivity of metabolism and ventilation (***E***_***o***_ = 0.35 eV at 15 °C with ∂***E***_***o***_/∂T = 0.01 eV/°C), while the second model includes metabolism but not ventilation (‘Metabolism model,’ dashed gray line; *E*_*d*_ = 0.52 eV). No allometric effect is included in either model (***ε*** = 0). (**B**) The Δ^13^C_oto_ of otolith observations relative to the temperature-dependent model (with ***ε*** = 0) for wild (red dots) and reared (black dots) Atlantic cod (data from^30^ [wild and reared], ^31,32^ [wild], ^19^ [reared]), plotted against body mass. Model curves are plotted for the net allometric dependence of metabolism and ventilation (‘Hypoxia,’ solid black curve; mass exponent −***ε*** = 0.06, predicting a small decrease in Δ^13^C_oto_ with mass) and for the allometric dependence of metabolism only (‘Metabolism,’ dashed gray curve; mass exponent ***δ***−1 = − 0.18, predicting a moderate increase in Δ^13^C_oto_ with mass). Both allometric models are referenced to 250 g, near the median mass of the four datasets plotted. (**C**) Distributions of ***SMS*** estimated using measured isotopic compositions, grouped by wild (red histogram and fitted probability distribution, n = 49) and reared (black histogram and fitted probability distribution, n = 98) Atlantic cod (assumed $\text{P}_{\text{w}}^{\text{C}}$ = 370 μatm and ***V***_***h***_ = 0.062 atm at 15 °C; ^4^). The independently, biogeographically inferred mean ***Φ***_***c***_ (species level estimate of ***SMS***) for wild cod is 2.4 (^4^).

The δ^13^C_oto_ of Atlantic cod decreases with rising temperature, a trend seen across many species^33^ and reflecting multiple chemical and biological factors (Fig. 3A). Aragonite enrichment relative to CO_2(aq)_ linearly decreases as temperature increases (Δ^13^C_A_ decreases by − 1.2‰ for every 10 °C of warming^34^). This is partially offset by the increasing δ^13^C_w_ with warming of the CO_2_ entering the gills (Eq. 1). Other chemical factors—gas solubility and diffusion and resulting variation in Q(T), and minor contributions of pH and carbonate system speciation—are smaller but reinforce the fractionation-driven trend towards decreasing δ^13^C_oto_ with increasing temperature. Cumulatively, these modeled chemical processes can account for roughly half of the observed temperature sensitivity of δ^13^C_oto_ in Atlantic cod.

The decline in δ^13^C_oto_ with temperature due to chemical factors (primarily isotopic fractionation) may be enhanced by physiological factors. For most species, including Atlantic cod, metabolism and ventilation both increase with temperature. The thermal acceleration of metabolic rate exceeds that of ventilatory response, resulting in a $\text{P}_{\text{met}}^{\text{O}}$ (and $\text{P}_\text{met}^\text{C}$) that rises with temperature (***E***_***o ***_> 0). The associated increase in the internal burden of metabolic CO_2_ causes a declining δ^13^C_oto_ trend that reinforces that from fractionation, resulting in an overall temperature dependence that matches observations. Because ventilatory flushing of CO_2_ partially offsets the rise in metabolic rate (cod ***E***_***o***_ is ~50% of the metabolic exponent, *E*_*d*_), it yields a weaker net impact on δ^13^C_oto_ than would result from metabolism alone (Fig. 3A, solid line). This ventilatory compensation of metabolism is typical of marine ectotherms, and provides an explanation for the observation that the temperature sensitivity of δ^13^C_oto_ is overestimated by metabolic scaling^19^ (Fig. 3A, dashed line).

Both isotope fractionation and metabolic effects yield an approximately linear relationship between temperature and δ^13^C_oto_, whereas the relationship between temperature and observed δ^13^C_oto_ reveals a slope that steadily increases as temperatures rise. This curvature is also predicted by hypoxia traits, in particular the tendency of ***E***_***o***_ to increase with temperature. This phenomenon arises because the temperature dependence of ventilation is stronger in cooler waters and weakens in warmer conditions as diffusion limits O_2_ supply, such that ***E***_***o***_ approaches *E*_*d*_^16^. When incorporated into the otolith model, the increase in ***E***_***o***_ with temperature reflects a weakening role of ventilatory compensation so that the temperature dependence of δ^13^C_oto_ in warmer waters converges on that of metabolism alone. The non-linearity in δ^13^C_oto_ predicted from hypoxia traits is also consistent with the curvature in the empirical data, which is much better fit by the model based on temperature-dependent hypoxia (***E***_***o***_) than a model based on temperature-dependent metabolic rate (*E*_*d*_) (Table 2).

While temperature is a key driver of respiratory physiology, the role of body size is debated^35,36^. Empirically, the hypoxia vulnerability of fishes varies much less with body size than with temperature^26^, leading to a typically small allometric scaling (***ε*** ~ − 0.05). This small magnitude of ***ε*** results from a close coupling of size-dependent rates of metabolism and ventilation; while metabolic rates increase with size as approximately B^0.8^ (or mass normalized ***B***^(0.8−1)^), ventilation rates also increase with size as approximately B^0.75^^8,36^. Atlantic cod again illustrate the concordance of observed δ^13^C_oto_ with these hypoxia traits (Fig. 3B). After removing the temperature dependence, wild and reared cod δ^13^C_oto_ (and resulting estimates of $\text{P}_{\text{met}}^{\text{O}}$) lack a significant trend with mass (Table 2).

The small trend in δ^13^C_oto_ with body size in wild and reared Atlantic cod is consistent with the measured net allometry of metabolism and ventilation in this species (mass normalized exponent −***ε*** = 0.06(se 0.15), for 500–2000 g fish;^37^). In contrast, observations of cod δ^13^C_oto_ do not support either the sign or magnitude of the strong increase in δ^13^C_oto_ with mass expected from the experimentally measured mass dependence of metabolism alone (exponent ***δ***−1 = − 0.18(se 0.04) from^38^; Fig. 3B). While ***ε*** varies with mass within and across species^8^, any such trends appear to be weak compared to other sources of variation in otolith δ^13^C_oto_, such as activity level (as hypothesized in^39^).

The ratio of sustained to resting metabolic rate in the Atlantic cod datasets reveals a clear difference between the reared and wild animals (Fig. 3C; *SM*, Table S2). Reared fish^19,30^ have δ^13^C_oto_ matching model predictions for resting cod (mean ***SMS ***= 1.0(se 0.05)) based on prior respirometry^40^. The higher values of wild cod (mean ***SMS*** = 2.6(se 0.1) are similar to independent, species-wide estimates inferred from biogeographic distributions (***Φ***_***c***_ = 2.4)^4^. The ***SMS*** derived from the otoliths of reared cod implies that their metabolic rates are both smaller and less variable than active energetic demands of animals in natural environments.

Based on the physiological model, the amount of metabolic carbon in the cod is sensitive to variations in metabolic rate at low ***SMS*** typical of resting fish in rearing studies (as described in^19^), but much less so at the higher ***SMS*** typical of active fish in natural environments. This is because most internal carbon is already metabolically derived when metabolic rates are high (Fig. 1B; δ^13^C_met_ reflects the diet plus any trophic enrichment, *SM*, Text S2.4). Therefore, δ^13^C_oto_ from wild fish is expected to be more sensitive to the magnitude of δ^13^C_met_ and variations in the diet (as proposed by^17,41^) than to differences in metabolic rate between individuals of the same species. Activity and diet driven differences in δ^13^C_oto_ are both found in the Atlantic cod datasets (*SM*, Fig. S3). In contrast to otolith sensitivity to diet and activity (and in contrast to prior expectations, e.g.^29^), this model predicts that δ^13^C_oto_ is insensitive to variations in δ^13^C_w_—except indirectly through the composition of primary production propagated up the food web—because usually $\text{P}_{\text{w}}^{\text{C}}$ << $\text{P}_{\text{met}}^{\text{C}}$ (Fig. 1B and *SM*, Fig. S3).

### Life-history variability

Individual otoliths contain seasonal to interannual growth bands with the potential to resolve isotopic fluctuations related to environmental and metabolic processes. The physiological model provides a means to predict and test such variations within individuals. A key observational trend is that as teleost fish develop, otolith δ^13^C_oto_ is often depleted in early life stages compared to adult values, followed by strong enrichment towards an adult value with annual or longer period oscillations^19,30,42,43^.

This trend was recently interpreted as driven by the reduction in intrinsic (per mass) metabolic rate^19^. However, because δ^13^C_oto_ depends on the ratio of metabolic to ventilatory rates, and the latter increases more slowly with size, the contribution of metabolic carbon should increase, not decrease, with size. Thus, changing body mass would require an allometric exponent far outside the range experimentally determined in fish and other marine organisms^8^ to explain individual δ^13^C_oto_ trends.

**Fig. 4:**
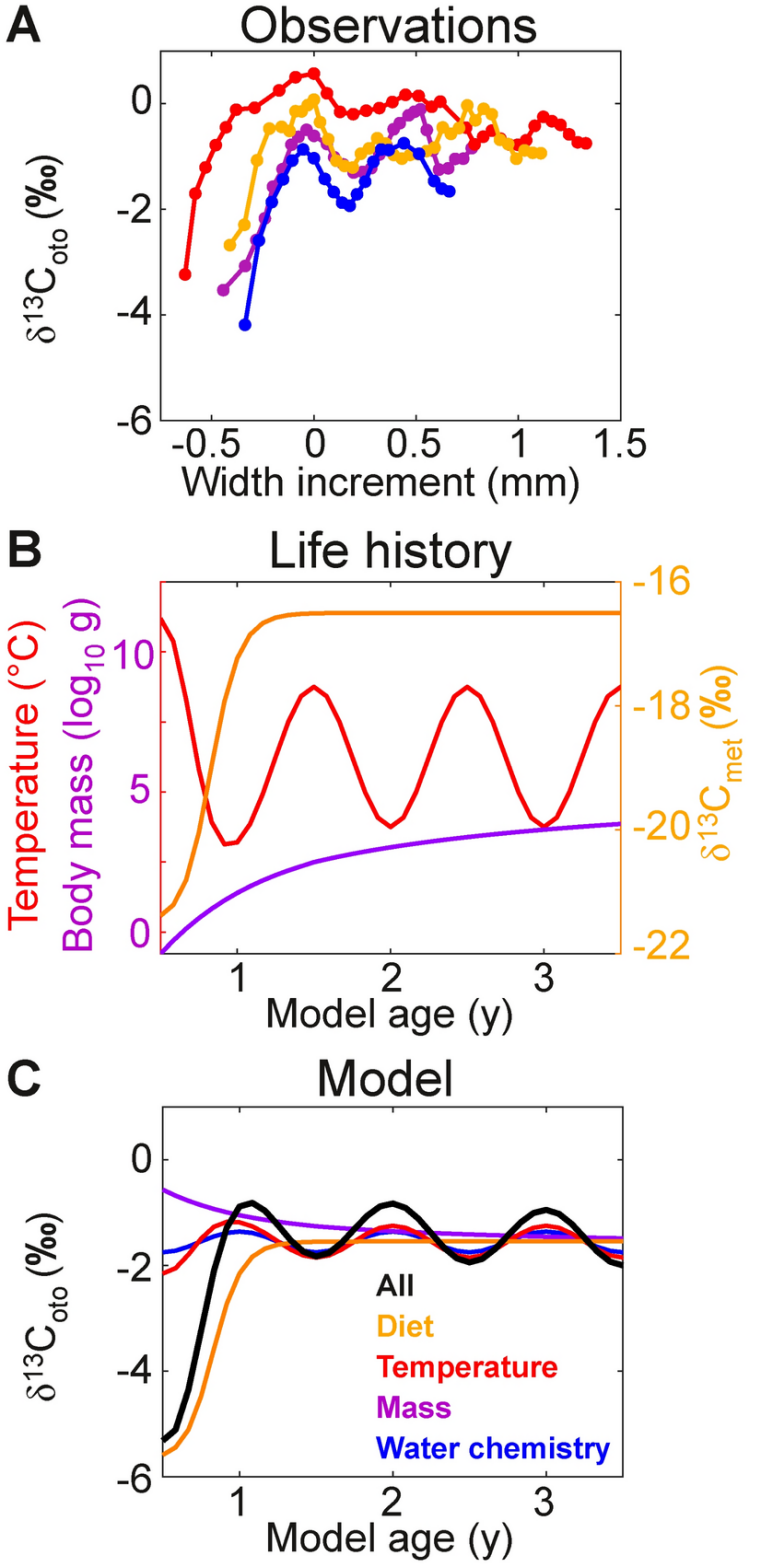
Comparison of ontogenetic δ^13^C_oto_ trends observed for Pacific cod and modeled for idealized life history scenarios. (**A**) Measured δ^13^C_oto_ from representative samples, as width increment along the otolith radius, relative to the first minimum temperature ^18^O/^16^O peak (from ^43,44^). (**B**) Idealized model forcing for life history changes in temperature (red), body mass (purple), and diet (δ^13^C_met_; orange) between post-flexion larvae and adult characteristics and with seasonal environmental cycles (*SM*, Text S4), from 6 months of age onwards. The plotted seasonal temperature cycle includes surface associated conditions for post-flexion larvae and migration to depth of older juveniles and adults. (**C**) Idealized model response of δ^13^C_oto_ to selected forcings, including: seasonal changes in water chemistry (blue), specifically pH (7.7–8.0) and $\text{P}_{\text{w}}^{\text{C}}$ (350–450 μatm); body mass (purple) via the net allometric dependence (mass exponent − ***ε*** = 0.1); temperature (red) changes via the net physiological temperature dependence (***E***_***o***_ = 0.35 eV at 4 °C with ∂***E***_***o***_/∂T = 0.01 eV/°C); shift in diet (orange) from plankton to larger invertebrates (δ^13^C_met_ = − 21.5‰ to − 16.5‰); all variations (full hypoxia model; black) with season and life stage. For all models, ***SMS*** = 2.5, and ***V***_***h***_ = 0.05 at 4 °C.

We examined δ^13^C_oto_ variations over the lifespan of Pacific cod (*Gadus macrocephalus*; Fig. 4A), using a dataset collected in the Bering Sea and sampled with unusually high temporal resolution (weeks to months^43,44^) that reveals both ontogenetic and seasonal patterns. In order to evaluate the source of age-related changes in δ^13^C_oto_, we generated plausible scenarios for the life-history changes in carbon sources and environmental conditions between post-flexion larvae, which live in surface waters and feed primarily on plankton, and deeper-living adults, which feed on invertebrates and fish (Fig. 4B; variable estimation described in *SM*, Text S4). These scenarios are used to simulate the time series of δ^13^C_oto_ generated incrementally over an individual’s lifespan using representative hypoxia trait values for gadid fishes based on the interspecific compilation^4^.

The shift from a planktonic diet to higher trophic level foods is essential to generate the correct magnitude and timing of otolith isotopic enrichment (Fig 4C). This finding is consistent with prior work demonstrating that changing δ^13^C_oto_ is associated with diet shifts during ontogeny, between habitats, and in controlled experiments^17,41,45–47^. The concurrent deepening of habitat into cooler waters reinforces the trophic shift. Temperature is also predicted to drive reinforcing trends in both $\text{P}_{\text{met}}^{\text{C}}$ and $\text{P}_{\text{w}}^{\text{C}}$, leading to consistency between modeled and observed seasonal oscillations of δ^13^C_oto_ around the mean adult composition.

While activity changes can readily explain differences between individual records (Fig. 4A), as previously with Atlantic cod (Fig. 3C) and across species^39^, systematic ***SMS*** shifts with life stage are not required or sufficient to generate plausible modeled δ^13^C_oto_ time series. Large changes in activity might be expected with the transition from yolk-based nutrition to feeding and from cutaneous respiration to gill-breathing^48,49^, but otolith records are difficult to resolve during these earliest life stages.

The inferred relative importance of the above drivers of within-otolith variation (diet, temperature, water chemistry, and mass) may be generalizable to varied teleost fishes with divergent early life stage and adult diets and environmental preferences. For example, mesopelagic orange roughy (*Hoplostethus atlanticus*) have initial isotopic enrichment and oscillations that span decades rather than seasons^42^. Yet the timing and degree of these δ^13^C_oto_ variations also qualitatively align with life history driven changes in temperature and diet. In contrast, rearing experiments maintaining similar diets and temperatures tend not to produce early life stage to adult δ^13^C_oto_ enrichments with similar magnitude or timing^30,48,50^, reinforcing the importance of changes in diet and habitat over an organism’s life-history in otolith records.

### Global variability

We simulated global distributions of δ^13^C_oto_, across species and environments, in order to evaluate the role of spatial variation of hypoxia traits in driving otolith patterns between biomes. Observations of δ^13^C_oto_ across all species remain too sparse to map detailed geographic distributions. However previous data compilations have revealed a significant trend toward declining δ^13^C_oto_ at higher temperatures (e.g.^33^). Such trends provide a reduced-dimension test of our trait-based model’s applicability at global scales. We simulated the spatial distribution of δ^13^C_oto_ accounting for variability in species hypoxia traits, environmental conditions, and dietary carbon sources (see Methods and Materials and *SM*, Text S5, Figs. S4 and S5). For this analysis, modeled δ^13^C_oto_ represents long-term mean, depth-averaged conditions and corresponding adult otolith compositions—the output is meant to illustrate major trends rather than resolve specific life-histories as in the previous analyses.

**Fig. 5:**
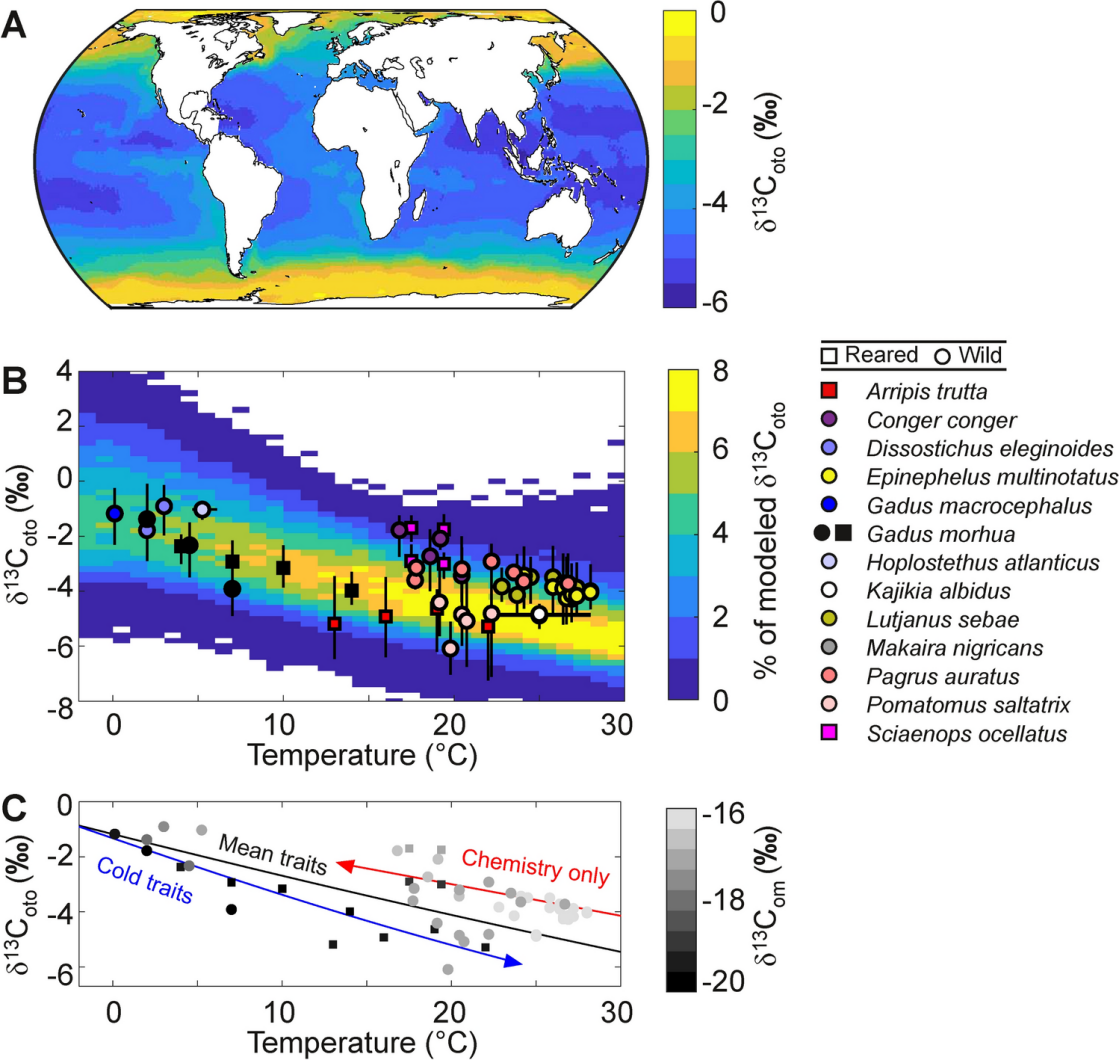
Global distribution and temperature dependence of modeled upper ocean δ^13^C_oto_. Model fields are simulated across the observed distributions of hydrographic conditions, hypoxia traits, and fish trophic levels (described in *SM*, Text S5), averaged over the top 200 m of the water column. (**A**) Spatial variations in modeled δ^13^C_oto_, driven by the spatial pattern of hypoxia traits that are viable at each location under climatological T and pO_2_^6^. (**B**) The resulting δ^13^C_oto_ from the global surface ocean across the range of ocean annual mean temperature. Shading indicates the percent of modeled parameter combinations (from hypoxia trait, environmental carbon, and diet distributions) with δ^13^C_oto_ at each temperature (each temperature bin sums to 100%, with more commonly modeled δ^13^C_oto_ shaded yellow and less common δ^13^C_oto_ shaded blue). Literature values of observed δ^13^C_oto_ from select species are overlaid (circles and bars are mean and range, see legend; *SM*, Table S2). Individual species have trophic levels (diets) that vary within the modeled range. Observational datasets include either juvenile or adult animals; one wild cod dataset includes both, with the youngest animals (black circle at ~ 7 °C) having more depleted tissue and otolith compositions^31,32^. (**C**) The same species observations shaded by measured or estimated organic matter composition, δ^13^C_om_ (approximate δ^13^C_met_; *SM,* Text S5.1) compared to model solutions (curves) for specific hypoxia traits and diet compositions, including: the mean δ^13^C_oto_ resulting from the mean of all measured hypoxia traits and mean δ^13^C_om_ of the plotted species (‘Mean traits,’ black), the means for just cold-adapted communities found at or below 5 °C (‘Cold traits,’ blue), and the temperature dependence of isotopic fractionation and other chemical factors (‘Chemistry only,’ red) without any net biological temperature effect (***E***_***o***_ = 0 eV).

Otolith carbon isotopic compositions predicted from distributions of environmental parameters and hypoxia traits are consistent with observed global trends in δ^13^C_oto_ across biomes and species (Fig. 5). Modeled community-mean δ^13^C_oto_ is most commonly between − 7 and + 2 ‰, with the most depleted values in the warm subtropical gyres and more enriched values in the cool polar oceans and upwelling regions (Fig. 5A; averaged from 0 to 200 m). The model also captures key features of the observed interspecific trend in whole-otolith δ^13^C with temperature (Fig. 5B), including the moderate negative slope (roughly − 1.5‰ with each 10 °C warming) and typical range of otolith compositions (a few ‰) at a given temperature.

That broad trend is modified by the variation of hypoxia traits across biomes^5^, which causes community δ^13^C_oto_ to decrease more with warming than expected from chemistry alone (Fig. 5C, in which the black line represents the mean hypoxia traits across species tabulated in^4^). The δ^13^C_oto_ decrease with temperature at the species level caused by ***E***_***o***_, Δ^13^C_A_(T), and Q(T) is enhanced by greater active hypoxia vulnerabilities (***V***_***h***_ · ***SMS***) in cool-water animal communities (Fig. 5C, in which the blue line represents the mean traits across species found at ≤ 5 °C; *SM*, Fig. S5). There is a shift toward species with lower and less temperature-dependent ***V***_***h***_ · ***SMS*** as well as in warmer ocean regions^5^ (Fig. S5), which may be an adaptive response to strong vertical habitat constraints imposed by shallower and more intense tropical oxygen minimum zones^51^. The effect of this biome-scale transition is to reduce the net biological response to increasing temperature, and above ~20 °C the slope is well approximated by the temperature-sensitivity of inorganic carbonate system fractionations alone (Fig. 5C red line).

Beyond this global-scale variation across temperature, at any fixed temperature otolith measurements from species with more isotopically enriched tissues also tend to have greater δ^13^C_oto_ than the modeled community mean, while those with more isotopically depleted tissues tend to have lower than the average modeled δ^13^C_oto_ (Fig. 5C). In other words, diet appears to be a primary driver of δ^13^C_oto_ differences between species living in otherwise similar environmental conditions, as within organisms and across populations.

## Predicting hypoxia traits from otoliths

The ability to predict δ^13^C_oto_ from quantitative hypoxia traits and environmental parameters (Figs. 3 and 4) provides a critical test of the model. However, its promise lays in reversing those inferences to derive key hypoxia traits—hypoxia vulnerability and its temperature and body size dependences—from more abundant and easily measured δ^13^C_oto_, especially for species that have not (or cannot) be established experimentally. We used δ^13^C_oto_ to predict the hypoxia vulnerabilities of fish species, by rearranging Eq. (1) to solve for $\text{P}_{\text{met}}^{\text{C}}$ and then the equivalent $\text{P}_{\text{w}}^{\text{O}}$. We compared otolith-derived $\text{P}_{\text{w}}^{\text{O}}$ to estimates derived from laboratory respirometry and biogeographic distributions. This calculation is described in the trailing Methods and Materials, and detailed in the supporting materials (*SM*, Text S6 and Table S3).

**Fig. 6:**
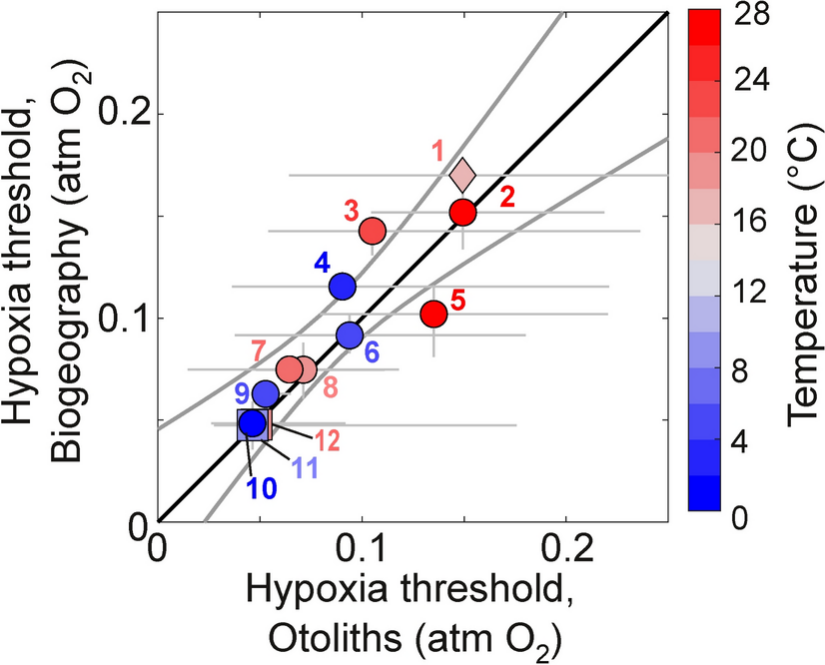
Comparisons of hypoxia thresholds (as $\text{P}_{\text{w}}^{\text{O}}$) calculated from otolith composition (‘Hypoxia threshold, Otoliths,’ x-axis), using the physiological model, with independent estimates from oxygen-based methods (‘Hypoxia threshold, Biogeography,’ y-axis). Observed species distributions are used to identify the lowest inhabited $\text{P}_{\text{w}}^{\text{O}}$ at the same environmental temperature (symbol face color) as the animals sampled for otoliths; these biogeography-derived estimates represent active organisms in natural environments (circles) and are compared to otolith-derived estimates from wild-caught animals. Two plotted datasets measure the hypoxia threshold using respirometry on resting organisms reared in experimental settings (squares), and are compared to otolith-derived estimates from reared animals. One experiment used wild caught animals that were subsequently reared for a short duration (diamond), and otolith-derived estimates from the temporarily reared animals (representing some combination of resting and active states) are compared to the biogeographic hypoxia threshold (for active organisms). In all cases, the oxygen- and otolith-derived hypoxia thresholds are calculated from independent observational datasets. Horizontal bars indicate the range of individual otolith-based estimates. Vertical bars indicate the range of oxygen-based estimates calculated from the hypoxia temperature sensitivity across the reported temperature range of the otolith samples. Species plotted include (*SM*, Table S2): (1) *Arripis trutta* (wild caught then reared), (2) *Epinephelus multinotatus*, (3) *Pomatomus saltatrix*, (4) *Dissostichus eleginoides*, (5) *Lutjanus sebae*, (6) *Gadus morhua*, (7) *Pagrus auratus*, (8) *Conger conger*, (9) *Hoplostethus atlanticus*, (10) *Gadus macrocephalus*, (11) *Gadus morhua* (reared), (12) *Sciaenops ocellatus* (reared). Note that Atlantic cod (*Gadus morhua*) has separate comparisons for reared and wild data. The black line represents a 1:1 correlation and the curved gray lines represent the 95% confidence interval of the linear regression (Table 2) through the species-specific means of all datasets.

Hypoxia thresholds derived from respirometry agree well with those from otoliths from reared populations (Fig. 6; Table 2). While comparisons to direct experimental values are extremely data limited, the consistency between the model and observations is also uniquely valuable. For reared fish with controlled diets and seawater conditions, otolith estimates integrate over time scales not possible with short-term laboratory respirometry. The relative ease of measuring otolith isotopic compositions also permits evaluation of hypoxia traits across many more organisms than is typically feasible in experiments.

For wild populations, temperature-dependent hypoxia traits have been shown to predict the range boundaries of many species^4,11,52^. For species with well-known biogeographic distributions, the inhabited combinations of temperature and pO_2_ define an aerobic niche in which the minimum inhabited pO_2_ corresponds to the active hypoxia threshold^5^. Because geographic distributions are available for most species with otolith δ^13^C_oto_, we compared the active hypoxia threshold in wild animals estimated from otolith-based geochemistry to those inferred from biogeographic niche mapping, at the measured otolith temperatures. As with reared animals and resting hypoxia thresholds, otolith compositions also generally predicted with high fidelity the hypoxia thresholds identified from biogeographic limits (Fig. 6; Table 2).

The strong correspondence between otolith and both experimental and biogeographic hypoxia thresholds supports the use of a mechanistic otolith model to extract key hypoxia traits. Moreover these results suggest that otoliths interpreted through this model may directly reveal biogeographic range limits governed by those hypoxia traits.

## Discussion and conclusions

The close coupling of O_2_ and CO_2_ in animal respiration yields a fundamental link between the carbon isotopic ratios of fish otoliths and their hypoxia vulnerability. The mechanistic model shows skill in predicting δ^13^C_oto_ patterns across multiple scales, from individuals to biomes, when hypoxia traits are known. It also demonstrates skill in predicting the reflection of those traits in the aerobic limits that bound species respiratory needs in the laboratory and geographic ranges in the ocean. Otoliths thus show substantial new promise not only in estimating hypoxia traits but also in the direct determination of aerobic habitat boundaries of marine fish species.

The model approach used here can be readily extended to a broader set of hypoxia traits, including the sensitivity of hypoxia vulnerability to temperature and body size. In principle, such estimates can be applied to archival and fossil otoliths to estimate hypoxia traits spanning historical and paleoceanographic conditions. Moreover, otolith-based trait estimates would provide rare and valuable quantification of individual hypoxia trait variability within a population, which may be substantial. Here we outline the potential extensions of the model that new and more comprehensive datasets would enable.

The thermal sensitivity of hypoxia vulnerability (***E***_***o***_) can be inferred from δ^13^C_oto_ measurements across a wide range of temperatures and species. Moreover, as illustrated by Atlantic cod, non-linearity in δ^13^C_oto_ records the transition from lower ***E***_***o***_ in cold water, where the compensation between metabolism and ventilation is strong, to higher ***E***_***o***_ in warm waters converging on the value from metabolism alone (Fig. 3A). Such trends are poorly resolved by historical respirometry, yet have important implications for the potential role of hypoxia in limiting the cold edge of species ranges^16^ and the magnitude of habitat compression with ocean warming.

Time-resolved δ^13^C_oto_ data could generate novel estimates of ***V***_***h***_ across life stages at an individual scale. Measurements of ontogenetic changes in hypoxia vulnerability at a species level are key to understanding the potential adaptive response of marine animal body sizes to ocean warming and oxygen loss^8^, but estimates of the small allometric exponent (***ε***) are scarce and likely clouded by variation among individuals. Age and size-resolved δ^13^C_oto_ variations in reared populations with controlled temperature and dietary effects would yield more precise estimates of ***ε*** for many species (e.g.,^30^). A better accounting for ontogenetic effects would also allow life history changes in wild populations to be more clearly revealed (Fig. 4A).

Global and biome scale patterns of δ^13^C_oto_ could provide valuable constraints on adaptation and selection of hypoxia traits. Otolith-based estimates of hypoxia vulnerability could be used to test for local adaptation in traits across the geographic range of species, provided variations in dietary and seawater inputs are also accounted for. Isotope thermometry from the same otoliths (e.g., oxygen or carbonate clumped isotopes^53,54^) may be a particularly powerful pairing, with the potential to reveal behavioral regulation of organism temperature in changing environments^55,56^. Dietary inputs may also be resolvable from protein collected from the same otoliths^46,57,58^.

At a global scale, our results imply that community-level variation in active hypoxia vulnerability, ***V***_***h***_ **·** ***SMS***, contributes significantly to the relationship between δ^13^C_oto_ and temperature across species (Fig. 5B). The corresponding gradients in active hypoxia vulnerability across latitude have been identified as a key factor driving biogeographic responses to climate, including selectivity of extinction^6,15^. Otolith measurements across sufficient species would provide independent tests of such latitudinal adaptation in hypoxia vulnerability among ecological communities, and could provide a tool to leverage archaeological and paleoceanographic otolith records to assess adaptations to past climates.

## Methods and materials

The development of the physiological model relating hypoxia traits and internal carbon pools is described heuristically in the Model section of the text, resulting in Eq. (2). The associated oxygen mass balance has been previously described in detail^26^. Similarly, while centered here on organismal fluxes, the underlying carbon mass and isotope balances are functionally equivalent to those derived for the surface ocean with respect to ecosystem-scale metabolism and ventilation^59,60^. The detailed derivation combining these mass balances and including known isotopic fractionations is presented in the *SM*, Text S1. Literature sources of variable estimates and parameterizations, as well as associated assumptions are noted in the *SM*, Text S2. The blood carbon measurements used to validate the relationship between oxygen and carbon-based parameter estimates are described in the supplementary materials (*SM*, Text S2 and compiled in Table S1).

Simulated δ^13^C_oto_ timeseries tracking the life-history of Pacific cod substitute typical hypoxia traits based on two previously compiled databases of many species^4,8^ for the unknown traits of this species (*SM*, Text S2 and Text S4). Changes in diet and environment between post-flexion larval and adult fish are estimated from literature sources, as are typical annual-mean and seasonal variations in environmental conditions in the Bering Sea that contribute to solutions of Eq. (1). Specific references and assumptions are detailed in the supplementary materials (*SM*, Text S4), and may be generalizable to species with similar life-history and habitat constraints.

The global estimation of δ^13^C_oto_ uses the same calculations as the species-specific analyses, with the exception that modeled physiological parameters are drawn from the full distributions of hypoxia traits (***E***_***o***_, ***V***_***h***_, and ***SMS***) from the previously compiled databases^4,8^, locally excluding only those trait combinations that cannot be supported by hydrographic conditions at the same location (e.g., ***V***_***h***_ · ***SMS*** adjusted by physiological temperature-dependence ***E***_***o***_ is greater than ocean pO_2_). This approach has been shown to reproduce both the latitudinal pattern of those traits and of species richness^6^. Local hydrographic conditions are estimated using climatological fields from the World Ocean Atlas^61^, and isotopic contributions from seawater from observational correlations between hydrographic parameters (temperature, salinity, phosphate concentration, and apparent oxygen utilization) and δ^13^C_DIC_ (*SM*, Text S5)^62^. The metabolic carbon isotopic composition is estimated as a trophic level-dependent difference between measured δ^13^C_om_ and the isotopic composition of local seawater (representing spatial variations in the isotopic composition of photosynthetically derived carbon at the base of the food web), calibrated to a compilation of literature estimates of environmental and tissue carbon for varied fishes and their trophic levels (*SM*, Table S2). Modeled metabolic contributions are estimated across the full trophic distribution of marine fish^63^. The combined distributions of hypoxia traits, trophic levels, and hydrography from these databases are used to generate weighted solutions of the range of otolith isotopic compositions expected in each water parcel and across the global ocean (*SM*, Text S5).

Estimates of hypoxia thresholds from isotopic datasets are based on a rearrangement of Eq. (1) in order to use measured isotopic values to estimate the ratio of $\text{P}_{\text{met}}^{\text{C}}$ / $\text{P}_{\text{w}}^{\text{C}}$ for individual samples, and conversion to the $\text{P}_{\text{w}}^{\text{O}}$ required in the environment (*SM*, Text S6.1). This metric is equivalent to the activity-dependent hypoxia vulnerability ***V***_***h***_ · ***SMS*** except that it is not normalized to a common reference condition (e.g., 15 °C). The otolith-derived estimates are compared to $\text{P}_{\text{w}}^{\text{O}}$ derived from the lowest oxygen associated with the observed presence of wild animals of the same species at the same temperature^5^ (*SM*¸ Table S3). In two datasets with reared fishes, the otolith-derived estimates are instead compared to $\text{P}_{\text{w}}^{\text{O}}$ from independent respirometry experiments with resting animals. This calculation, as well as that of ***SMS*** similarly estimated from isotopes, is sensitive to uncertainties in the underlying isotopic measurements. In particular, the estimate of $\text{P}_{\text{met}}^{\text{C}}$ / $\text{P}_{\text{w}}^{\text{C}}$ becomes unconstrained as δ^13^C_int_–δ^13^C_met_ decreases below ~0.7‰ (the 1-sd replicate uncertainty in the difference; *SM*, Text S6.2 and Fig. S6). We exclude results with δ^13^C_int_–δ^13^C_met _< 0.7‰ from Figs. 3C and 6.

## Supplementary Information

Supplementary Information.

Supplementary Table S1.

Supplementary Table S2.

Supplementary Table S3.

## Data Availability

All databases of ocean hydrography, fish trophic level, and hypoxia traits described in the methods and materials are publicly available (refer to citations). All other data are found in the main text or the *Supporting Materials*. Processed fields derived from the public databases as well as Matlab code used in this work are archived at https://github.com/EvanMHoward/Otolith13C_hypoxia.
