# Supplementary material for: Hypoxia traits imprinted in otolith δ13C from individual to global scales
Source: Sci Rep. 2025 Jan 2;15:279. doi: 10.1038/s41598-024-82518-0 (PMC11696016; doi:10.1038/s41598-024-82518-0)
Supplement: Supplementary file 1 — Supplementary Information. [file 41598_2024_82518_MOESM1_ESM.pdf]

Supplementary Materials for

Hypoxia traits imprinted in otolith δ13C from individual to global scales

Evan M. Howard and Curtis A. Deutsch

Email: ehoward2@uw.edu, cdeutsch@princeton.edu

This PDF file includes:

Supplementary Text S1: Derivation of otolith isotopic model
Supplementary Text S2: Physiological and chemical parameters used in isotopic model
Supplementary Text S3: Blood carbon measurements
Supplementary Text S4: Variable estimation for Pacific cod
Supplementary Text S5: Variable estimation for global analysis
Supplementary Text S6: Estimation of hypoxia traits from isotopic measurements

Figs. S1 to S5

Table S1: Measured and inferred internal carbon pools of Actinopterygid fish (external)
Table S2: Otolith isotopic data and metadata used in analyses (external)
Table S3: Hypoxia thresholds from otoliths and biogeography (external)
Table S4: Extended glossary of parameters used in the *Supplementary Materials*

Supplemental references

Supplementary Text S1: Derivation of otolith isotopic model

S1.1 Derivation

Here we derive an expression that relates otolith carbon isotope composition to hypoxia tolerance traits of a generic fish species. The derivation assumes that fluxes of total dissolved inorganic carbon (DIC) and oxygen (O_2) between an organism and its environment are balanced (inputs = outputs) and achieve an isotopic equilibrium among the aqueous carbon pools. To maintain the simplest possible model, both carbon and oxygen are characterized by a single internal concentration, which represents the fluid that accumulates and ventilates the products of metabolism. The model can be readily expanded to represent the isotopic differences between the blood and the endolymph (where the otolith is formed), should new observations become available to better constrain those carbon pools *.

The $^{13}C/^{12}C$ ratio (R) of otolith aragonite is related to that of the bicarbonate (HCO^{3-}) from which the aragonite is precipitated. In turn, the isotope ratio of bicarbonate is rapidly equilibrated with CO_2 through the standard aqueous CO_2 hydration reactions mediated by carbonic anhydrase. The isotopic equilibria can be written:

Eq. S1.1.1 $$R_{Arag} = R_{CO_2}\left(\frac{\alpha_{Arag/HCO3}}{\alpha_{CO_2/HCO3}(T)}\right)$$

The α terms are the known inorganic equilibrium speciation of stable isotopes for each reaction (*Text S.2.4*), and the overall fractionation factor is temperature-dependent. Thus, the otolith R_{Arag} can be computed from chemical equilibria once the isotopic ratio of internal CO_2 is known. While CO_2 is typically a small fraction of DIC, it is the carbon pool that exchanges with ambient water, and thus links the otolith to seawater chemistry (see below). In this text we use isotopic ratios for mathematical convenience, but convert the final solution to normalized values using standard notation, $\delta = (R / R_{standard} - 1) \cdot 1000$.

To predict the CO_2 and its isotopic ratio in internal fluids, we rely on the mass balance of organismal DIC:

Eq. S1.1.2 $$\frac{\partial(V \cdot DIC)}{\partial t} = \alpha_D^C - \alpha_S^C\left(P_{int}^C - P_w^C\right)$$

The left-hand side of Eq. S1.1.2 is the time rate of change of DIC integrated over the fluid volume (V), which is given by the difference between source and sink rates on the right-hand side. The first term on the right hand side is the source of DIC from respiration of organic matter at a rate α_D^C (mol C g^{-1} hr^{-1}) and the second term is the sink of DIC due to gas exchange of CO_2, whose rate is a product of a rate coefficient α_S^C (mol C g^{-1} atm^{-1} hr^{-1}) and the CO_2 partial pressure difference between internal fluid (P^C_{int}) and water (P^C_w). The use of α to denote rates (italicized) is chosen to emphasize the relationship to hypoxia traits (see below), and the similarity to chemical fractionation factors (Eq. S1.1.1) is an unfortunate coincidence of combining different notation conventions. Similarly, the subscripts on rate parameters α_D^C and α_S^C are chosen for

consistency with hypoxia traits, to denote supply (S, due to gas exchange) and demand (D, due to metabolism). An important feature of the organismal DIC budget is that the sink depends only on the fraction of DIC that is gaseous CO_2 †.

A similar equation for the ^{13}C component of DIC can be written by multiplying each carbon pool by the $^{13}C/^{12}C$ ratio of that pool, and each incoming flux by the $^{13}C/^{12}C$ of the relevant pool so that:

Eq. S1.1.3 $$\frac{\partial(\mathrm{V}\cdot{}^{13}\mathrm{DIC})}{\partial \mathrm{t}} = \mathrm{R_{met}}\cdot\alpha_D^C - \alpha_S^C\left(\mathrm{R_{CO2}}\cdot\mathrm{P_{int}^C} - \mathrm{R_w}\cdot\mathrm{P_w^C}\right)$$

Because the loss of DIC only depends on CO_2 pressure (P^C_{int}), the loss of ^{13}DIC also depends solely on the isotopic ratio (R_{CO2}) of the internal gaseous pool of CO_2. Similarly, during ventilation, ^{13}DIC is gained via the gills from CO_2 in the surrounding seawater with an isotope ratio R_w. Here R_{met} represents the isotopic ratio of DIC added to internal fluids from organic matter respiration (this is approximately the isotopic composition of the respired organic carbon from the diet, though the isotopic composition of the metabolic carbon entering the blood and other internal fluids is not necessarily that of the ingested bulk food or other fish tissues, see *Text S2.4*). The isotopic ratio R_{CO2} is directly related to that of aragonite precipitation in Eq. S1.1.1. Thus, substituting Eq. S1.1.2 and Eq. S1.1.3, into Eq. S1.1.1 yields an expression for R_{Arag} that is based on organismal rates of metabolism and gas exchange:

Eq. S1.1.4 $$\mathrm{R_{Arag}} = \frac{\left(\mathrm{R_{met}}\cdot\frac{\alpha_D^C}{\alpha_S^C} + \mathrm{R_w}\cdot\mathrm{P_w^C}\right)}{\left(\frac{\alpha_D^C}{\alpha_S^C} + \mathrm{P_w^C}\right)}\left(\frac{\alpha_{\mathrm{Arag/HCO3}}(\mathrm{T})}{\alpha_{\mathrm{CO_2/HCO3}}(\mathrm{T})}\right)$$

This expression is similar to previous diagnostic models in that the mineral R is a weighted sum of the isotopic ratios of water and metabolic end-members. However, there are three key differences in Eq. S.1.1.4 compared to prior representations: First, the expression explicitly accounts for the temperature-dependent chemical fractionation from dissolved carbon to the mineral phase. Second, the weighting of carbon masses is represented by a partial pressure of CO_2, not DIC. Third, the metabolic end member (P^C_{met}) is cast as a ratio of supply rate to the ventilatory flushing rate, both species-specific physiological rates that can in principle be independently measured. This last property is what makes the equation predictive rather than diagnostic.

The ratio of the two physiological rates, the metabolic source of carbon and its ventilatory sink, could in principle now be derived from experimental data. While there is a rich literature on metabolic rates and the dependence on body size and temperature to draw upon, little if any data exists for the coefficient of CO_2 gas exchange in water-breathing organisms. Instead, we turn to the organismal O_2 balance, where these rate coefficients can both be estimated from laboratory experiments. More importantly, the ratio of α_D / α_S, and its variation with temperature and body size can be directly determined, reducing the uncertainty arising from compiling separate estimates of the three related hypoxia traits (described below).

The O_2 balance of a general marine organism, can be written in an analogous fashion to that of DIC:

Eq. S1.1.5 $$\frac{\partial(\mathrm{V}\cdot \mathrm{O_2})}{\partial \mathrm{t}} = \alpha_S^O\left(\mathrm{P_w^O} - \mathrm{P_{int}^O}\right) - \alpha_D^O$$

The terms on the right-hand side represent the same processes as for DIC, but metabolism has become a sink and ventilation a net supply. The rate coefficients will differ in magnitude from the corresponding coefficients for C, and are thus distinguished by superscript ***O***. The balance in Eq. S.1.1.5 has been well studied in marine species. The ratio of rate coefficients can be estimated from the ambient O_2 pressure, P^O_w, below which internal O_2 (P^O_{int}) is too low to sustain resting metabolic rates (at this critical threshold, $P^O_w = P^O_{met}$). This is a measure of hypoxia vulnerability, which we denote $\boldsymbol{V_h}$ (atm) when referenced to a standard temperature and body mass, and it can be directly estimated from laboratory experiments. The ratio of O_2 supply to demand coefficients is observed to vary with body size, temperature, and activity level, as detailed in previous work[1]:

Eq. S1.1.6 $$\frac{\alpha_D^O}{\alpha_S^O} = \boldsymbol{V_h} \cdot \mathrm{B}^{-\boldsymbol{\varepsilon}} \cdot \exp\left(-\boldsymbol{E_o}/\mathrm{k_B} \cdot \mathrm{T}'\right) \cdot \boldsymbol{SMS}$$

The variables and hypoxia traits ($\boldsymbol{V_h}$, $\boldsymbol{E_o}$, $\boldsymbol{\varepsilon}$, and ***SMS***) are defined as in the main text and follow prior convention, with ***B*** representing the body mass normalized to a reference mass at which $\boldsymbol{V_h}$ is determined and $\boldsymbol{\varepsilon}$ the net allometric sensitivity of supply to demand, $\boldsymbol{E_o}$ (eV) is the net temperature sensitivity of demand to supply, k_B is the Boltzmann constant (eV K^{-1}), and T' is the inverse temperature difference from a reference temperature at which $\boldsymbol{V_h}$ is estimated ($T' = 1/T - 1/T_{ref}$, in K). The variable ***SMS*** represents the ratio of sustained to resting metabolic rate (or, if supply rate varies as well, the overall factor by which the active demand to supply rate ratio exceeds resting demand to supply rate ratio). When a species-level estimate of ***SMS*** is evaluated from biogeographic distributions, this term has previously been called $\boldsymbol{\Phi_{crit}}$ or $\boldsymbol{\Phi_c}$[2]. Empirical distributions of all four hypoxia traits based on prior work[1,3] are plotted in **Fig. S1**.

The rate coefficients for carbon can be related to those of O_2 through the following stoichiometric and kinematic relationships:

Eq. S1.1.7a $$\mathrm{R_Q} = \alpha_D^C/\alpha_D^O$$
Eq. S1.1.7b $$\mathrm{V_Q} = \alpha_S^C/\alpha_S^O = \frac{\mathrm{D_C(T)\cdot K_C(T)}}{\mathrm{D_O(T)\cdot K_O(T)}}$$
Eq. S1.1.7c $$\mathrm{Q(T)} = 0.5 \cdot \mathrm{R_Q}/\mathrm{V_Q}$$

R_Q is the respiratory quotient, i.e., the carbon to oxygen stoichiometric ratio of metabolism, and V_Q is the ratio of ventilatory rate coefficients, which depends on the temperature-dependent product of the diffusivity (D, m^2 hr^{-1}) and solubility (K, mol m^{-3} atm^{-1}) coefficients of each gas during exchange across the gills (the ratio of permeabilities). The P^O_{met} deficit at the gills derived from respirometry is twice the average across the circulation (see *Text S2.1* below), so the factor of 0.5 converts P^C_{met} inferred from respirometry to the internal average for this single reservoir model. The form and value of Q(T) are discussed in further detail in *Text S.2.2* (below).

Substituting Eq. S1.1.6 and Eq. S1.1.7c into Eq. S1.1.4, and applying the relationship between R and δ, yields the equation for $\delta^{13}C_{oto}$:

Eq. S1.1.8 $$\delta^{13}C_{oto} = \frac{\delta^{13}C_{met}[Q(T) \cdot \boldsymbol{V_h} \cdot B^{-\varepsilon} \cdot \exp(-\boldsymbol{E_o}/k_B \cdot T') \cdot \boldsymbol{SMS}] + \delta^{13}C_w \cdot P_w^C}{[Q(T) \cdot \boldsymbol{V_h} \cdot B^{-\varepsilon} \cdot \exp(-\boldsymbol{E_o}/k_B \cdot T') \cdot \boldsymbol{SMS}] + P_w^C} + \Delta^{13}C_A$$

The term in square brackets is the pressure of metabolic carbon in internal fluids (P^C_{met}), expressed in relation to measurable hypoxia traits of a generic species. The last term on the right-hand side is the isotopic effect of temperature-dependent fractionation between CO_2 and aragonite in the otolith. A complete interpretation of Eq. S.1.1.8 is given in the text, where it was heuristically derived.

Notes:

* There is one published study identifying isotopic differences between fluid compartments of freshwater trout in a series of isotope enrichment experiments[4]. Those results identify large and variable isotopic differences between diet, blood, endolymph, and otolith composition, including blood DIC compositions that are substantially depleted relative to both ambient water and dietary carbon. While there are few results from aquatic organisms to compare to, the magnitude, variability, and direction of the reported fractionation effects from that study diverge from the consistent, small fractionations found in other vertebrates (see review[5]). Such work has significant methodological and analytical challenges in water-breathing organisms. For example, while ideally the blood sampling approach should represent the whole DIC pool, blood was taken by cardiac puncture (representing a particularly high pressure and high carbon endpoint of the blood circulation) and the reported values appear similar to expectations for the CO_2 component alone (a decrease of several ‰ in $\delta^{13}C_{int}$). Similarly, there may be a significant non-steady state component to uptake of isotopically spiked carbon in the trout experiments. Given the paucity of data and challenges in interpretation, it will require substantial additional data on the isotopic composition of the whole-fluid carbon pools of fish to validate and refine such results and to extend any findings to marine organisms prior to incorporation of additional fluid pools into otolith models.

† The carbon mass balance (Eq. S1.1.2) assumes that other sources of inorganic carbon exchange from the internal fluids, such as bicarbonate excretion in fish intestines[6], lead to only small decreases in P^C_{int} relative to the large metabolic and ventilatory fluxes. An additional model factor could be added if such fluxes outside the gills were sufficiently large and well constrained. For example, if intestinal excretion was set to 25% of the magnitude of the ventilatory flux, model solutions increase by ~0.5 ‰—though the temperature and mass dependencies that are the focus of this work are unaffected. Excretion of carbon that remains entirely within the digestive tract and is not incorporated into the blood CO_2 pool (e.g. dissolved and particulate organic carbon waste generated from food, ingested seawater DIC) does not affect this mass balance.

S1.2 Steady-state mass and isotopic balances

For reference, here we provide a set of oxygen and carbon mass and isotopic balances and other key relationships used in the derivation of the otolith model.

Oxygen balances

Eq. S1.2.1 $\alpha_D^O = \alpha_S^O(P_w^O - P_{int}^O)$ Steady state mass balance

Eq. S1.2.2 $P_{int}^O = P_w^O - P_{met}^O$ Pressure balance

Eq. S1.2.3 $P_{met}^O = \frac{\boldsymbol{\alpha_D^O}}{\boldsymbol{\alpha_S^O}} = \boldsymbol{V_h} \cdot B^{-\varepsilon} \cdot \exp(-\boldsymbol{E_o}/k_B \cdot T') \cdot \boldsymbol{SMS}$ Temperature and mass dependence

Carbon balances

Eq. S1.2.4 $\alpha_D^C = \alpha_S^C(P_{int}^C - P_w^C)$ Steady state mass balance

Eq. S1.2.5 $P^C_{int} = P^C_{met} + P^C_w$ Pressure balance

Eq. S1.2.6 $P^C_{met} = \frac{\alpha^C_D}{\alpha^C_S} = Q(T) \cdot P^O_{met}$ See *Text S2.2*

Isotopic mixing and fractionation expressions

Eq. S1.2.7 $\delta^{13}C_{oto} = \delta^{13}C_{int} + \Delta^{13}C_A$ Inorganic fractionation

Eq. S1.2.8 $\delta^{13}C_{int} \cdot P^C_{int} = \delta^{13}C_{met} \cdot P^C_{met} + \delta^{13}C_w \cdot P^C_w$ Isotopic extension of S1.2.5

Eq. S1.2.9 $\frac{P^C_{met}}{P^C_w} = \frac{\delta^{13}C_w - \delta^{13}C_{int}}{\delta^{13}C_{int} - \delta^{13}C_{met}}$ or $\frac{P^C_{met}}{P^C_{int}} = \frac{\delta^{13}C_w - \delta^{13}C_{int}}{\delta^{13}C_w - \delta^{13}C_{met}}$ Diagnostic ratio of carbon sources ‡

Solution forms

Eq. S1.2.10 $$\delta^{13}C_{int} = \frac{\delta^{13}C_{met}[Q(T) \cdot \boldsymbol{V_h} \cdot B^{-\varepsilon} \cdot \exp(-\boldsymbol{E_o}/k_B \cdot T') \cdot \boldsymbol{SMS}] + \delta^{13}C_w \cdot P^C_w}{[Q(T) \cdot \boldsymbol{V_h} \cdot B^{-\varepsilon} \cdot \exp(-\boldsymbol{E_o}/k_B \cdot T') \cdot \boldsymbol{SMS}] + P^C_w}$$

Eq. S1.2.11 $$\delta^{13}C_{oto} = \frac{\delta^{13}C_{met}[Q(T) \cdot \boldsymbol{V_h} \cdot B^{-\varepsilon} \cdot \exp(-\boldsymbol{E_o}/k_B \cdot T') \cdot \boldsymbol{SMS}] + \delta^{13}C_w \cdot P^C_w}{[Q(T) \cdot \boldsymbol{V_h} \cdot B^{-\varepsilon} \cdot \exp(-\boldsymbol{E_o}/k_B \cdot T') \cdot \boldsymbol{SMS}] + P^C_w} + \Delta^{13}C_A$$

Eq. S1.2.12 $$R_{oto} = \frac{R_{met}[Q(T) \cdot \boldsymbol{V_h} \cdot B^{-\varepsilon} \cdot \exp(\boldsymbol{E}-_{\boldsymbol{o}}/k_B \cdot T') \cdot \boldsymbol{SMS}] + R_w \cdot P^C_w}{[Q(T) \cdot \boldsymbol{V_h} \cdot B^{-\varepsilon} \cdot \exp(-\boldsymbol{E_o}/k_B \cdot T') \cdot \boldsymbol{SMS}] + P^C_w} \cdot \alpha_{Arag/CO_2}$$

Notes:

‡ Equation 1.2.9 differs from prior expressions for the ratio of metabolic to environmental (or alternatively, total) carbon in a key way: Consistent with the DIC mass balance presented above, the seawater and internal isotopic compositions of CO_2 are the relevant values, rather than the compositions of seawater and internal DIC. Note that the uncertainty in this ratio increases as the gap between the internal and metabolic compositions decreases towards zero. Thus the greater the ratio of P^C_{met}/P^C_w, the more sensitive it is to analytical errors or variations in the isotopic measurements of the dietary endmember and the otolith aragonite from which the internal carbon composition is estimated (*Text S6*, below).

Supplementary Text S2: Physiological and chemical parameters used in isotopic model

S2.1. Physiological parameters

The hypoxia vulnerability, $\boldsymbol{V_h}$, net temperature sensitivity of metabolism and ventilation, $\boldsymbol{E_o}$, and temperature sensitivity of metabolism alone, $\boldsymbol{E_d}$, are tabulated in the supporting information of Deutsch et al.[1]. The net allometric scaling of ventilation minus metabolism ($\boldsymbol{\varepsilon}$) is tabulated in the supporting information of Deutsch et al.[3].

One key difference between the treatment of $\boldsymbol{V_h}$ between this and prior works has to do with the internal fluid endmember P^O_{int}. By definition, $\boldsymbol{V_h} = \alpha^O_D / \alpha^O_S$ at a reference temperature and body mass, and $P^O_{int} = P^O_w - P^O_{met}$ where P^O_{met} is the partial pressure deficit generated by respiration (as opposed to CO_2, for which P^C_{met} represents a respiratory excess). In respirometry experiments used to derive $\boldsymbol{V_h}$, the critical partial pressure at which resting metabolism is exactly matched by supply occurs when $\alpha^O_D = \alpha^O_S \cdot (P^O_w - P^O_{int})$. At this extreme, $P^O_{int} = 0$ atm (all internal oxygen is consumed by metabolism) precisely at the end of the blood circulation and immediately prior to ventilation at the gills. Thus $\boldsymbol{V_h}$ is defined by the condition where the metabolic partial pressure deficit P^O_{met} is equal to P^O_w. If P^O_{int} reaches 0 atm before the end of the blood circulation, respiration must be suppressed below the resting metabolic rate and $P^O_w < \boldsymbol{V_h}$.

In contrast to the respirometric definition of $\boldsymbol{V_h}$ described above, the single reservoir model for bulk isotopic composition that we present in this work is expressed relative to average blood

partial pressures of oxygen and carbon. Thus the average P^{O}_{int} across the whole circulation should be half that in the ambient water ($[P^{O}_{w} + 0 \text{ atm}] / 2 = 0.5 \cdot P^{O}_{w}$).

Direct measurements of P^{O}_{int} are strongly dependent on the portion of the blood circulation being sampled, and are typically < 50% of P^{O}_{w} in the ventral blood vessels prior to gill ventilation and > 50% of P^{O}_{w} in the dorsal blood vessels following gill ventilation[7–12]. The average of pre- and post-ventilation blood samples is generally 40–60%.

The consistency between the theoretical expectation and experimental estimates of average P^{O}_{int} indicates that the value of $\boldsymbol{V_h}$ should be scaled by a factor of 0.5 to account for the different reference conditions (the maximum P^{O}_{int} and P^{O}_{met} deficits at the gills versus the average across the blood circulation) in order to generate an estimate of average P^{C}_{met} across the circulation for use in the isotopic model. Rather than use two different definitions of this term, we include the factor of 0.5 in the stoichiometric ratio used to convert between oxygen and carbon fluxes, Q(T).

S2.2. Stoichiometric quotient, Q(T)

The stoichiometric quotient Q(T) is a function of the CO_2/O_2 ratio of respiration relative to that of ventilation, i.e., $Q(T) = 0.5 \cdot R_Q / V_Q$ (where the factor of 0.5 is related to the $\boldsymbol{V_h}$ definition).

The flux of dissolved oxygen across a surface, such as a gill, can be described as $\partial[O_2] / \partial t = k_O \cdot K_{H,O} \cdot (P^{O}_{gas} - P^{O}_{w})$, where $K_{H,O}$ is the Henry's Law solubility coefficient and k_O is the gas transfer coefficient. The latter is proportional to diffusivity (D) as well as a function of the area and length scales of the gas exchange surface. As the size characteristics of the gills are unchanged when comparing CO_2 and O_2 exchange, the ratio of gas supply can be expressed in terms of diffusivity and solubility, $V_Q = (D_C \cdot K_{H,C}) / (D_O \cdot K_{H,O})$; in other words, the ratio of permeabilities. Diffusivity and solubility have different temperature sensitivities for each gas, leading to distinct rate coefficients for each gas and a temperature dependent ventilatory quotient on the order of 20.

The respiratory quotient, R_Q, is a molar ratio near 1. This value may increase with anaerobic metabolism and decrease with sustained aerobic exercise[13], presumably because fats ($R_Q \sim 0.7$) increasingly contribute to prolonged high metabolism. Differences in diet (i.e., proportion of carbohydrates, proteins, and lipids) could also lead to variations in R_Q. The R_Q of fish is generally not temperature-dependent (see review[14]). Throughout this work, the long-term mean R_Q contributing to otolith signals is assumed to be 1.

Combining the above considerations the stoichiometric quotient used in Equation 1 of the main text is calculated as $Q(T) = 0.5 \cdot R_Q / [(D_C \cdot K_{H,C}) / (D_O \cdot K_{H,O})]$.

The solubility coefficient and diffusivity for O_2 are calculated with the Hamme[15] ocean gas functions (using empirical equations of [16–18]). The effective CO_2 solubility coefficient (for combined CO_2 and carbonic acid) is calculated using the data of Weiss[19] as fit by Dickson et al.[20], and diffusivity from Zeebe[21], with salinity corrections in the same manner as the Hamme[15] ocean gas functions; this correction scheme does a good job matching observed salinity-

dependent diffusivity differences for a wide range of dissolved gases and inorganic solutes, including O_2, CO_2, and bicarbonate.

S2.3. Carbonate system parameters

Marine carbonate system equilibria in seawater, K_1' and K_2', are calculated from Millero[22], for use with the total pH scale. The effective CO_2 solubility coefficient $K_{H,C}$ is calculated as in *Text S2.2*.

Experimental carbonate system equilibria for freshwater trout blood[23,24] have been widely used in subsequent literature for calculating carbonate system parameters within the internal fluids of fish. These estimates lead to slightly higher partition coefficients in fish fluids than the equivalent parameters in fresh water, but lower than in salt water. When corrected for the effect on activity coefficients of ionic strength differences between water and fish fluids (in teleost fish, typically ionic strength is ~0.17 mol kg^{-1} and osmolarity ~300 mOsm regardless of ambient environmental salinity[6,25,26]), these estimates are indistinguishable from (within uncertainty bounds of) better studied seawater equilibria. For ease of calculation with existing seawater carbonate system equations as a function of salinity and temperature, fish fluids can be assumed to have an isoionic equivalent salinity of S = 9 as a good first order approximation of more complicated corrections for chemical activity.

Ocean pH is typically reported on the seawater or total pH scales, whereas physiological measurements are often reported on the United States National Bureau of Standards pH scale. The two can sometimes be interconverted using carbonate system equilibrium software like CO2Sys[27] and additional information about the pH electrode design and calibration and additional measurements of non-carbonate chemicals that influence alkalinity and acidity; however detailed information about chemical apparatus and assumptions is often missing from the physiological literature, so a reasonable rule of thumb is that $pH_{total} \sim pH_{NBS} - 0.15$ [28]. Internal fish fluid pH is not usually measured concurrently with isotopic composition, and the sensitivity of $\delta^{13}C_{oto}$ to the absolute magnitude of pH is small, so for this work pH_{total} in fish fluids is approximated as 7.7 (pH_{NBS} in fish fluids is typically measured as 7.7 to 8.0, refer to references in Table S1). The empirical temperature-dependence of pH is the same as that of the equilibrium coefficient of water at constant alkalinity, K_W, in both bulk ocean waters[29] and in fish blood[30]. Thus $\partial pH / \partial T = -0.01$ to -0.02 °C^{-1}, and is calculated here based on the temperature sensitivity of K_W[31] as in Dickson et al.[20].

S2.4. Isotopic fractionations

The temperature-dependent, inorganic equilibrium fractionation factors of the marine carbonate system are well-constrained (see review[32]). In this work, the fractionation factors between $CO_{2(g)}$ and carbonate, bicarbonate, and $CO_{2(aq)}$ are all calculated using the empirical fits of Zhang et al.[33] for internal consistency across experimental results. The temperature-independent inorganic isotope effect for the equilibrium fractionation between aragonite and bicarbonate is taken from Romanek et al.[34]. An important caveat is that the former experiments were conducted in seawater, and the latter determination was for synthetic, inorganic aragonite precipitated from solutions greatly diverging from the compositions of seawater, fish blood, or endolymph. While

the similar carbon chemistry in seawater and fish blood (see section *S2.3*) may support applications of the fractionations between dissolved carbonate system species, it is not clear whether the synthetic aragonite fractionation factor and it's assumed temperature-independence are applicable to otolith aragonite. The relationship between the composition of DIC (for example, in seawater) and other carbonate system components is solved algebraically using ionization fraction (α_i) expressions for the carbonate system[35]. The isotope effect between $\delta^{13}C_{int}$ and $\delta^{13}C_{oto}$, $\Delta^{13}C_A$, is calculated by combining both sets of empirical fractionation factors as shown in Eq. S1.1.1 and converting to isotopic delta notation. This equation requires prior appropriate conversion of other isotopic inputs to generate $\delta^{13}C_{int}$, including accounting for carbonate system differences between seawater and fish fluids.

Modeled $\delta^{13}C_{oto}$ for Atlantic cod better matches the magnitude of observed $\delta^{13}C_{oto}$ (typically around -3 ‰) if there is an additional constant enrichment of roughly +0.7 ‰ on $\Delta^{13}C_A$, or of +1 ‰ on $\delta^{13}C_{met}$ relative to the measured $\delta^{13}C$ of tissues measured concurrently with the otoliths from the same animals[36–38] or estimated from tissues of other animals from similar locations or experimental settings[39]. In this work, the latter parameter is included when estimating $\delta^{13}C_{met}$ for Atlantic cod from available tissue measurements. For the analysis of Pacific cod life history we assume the same increase because of its shared genus and similar life history, i.e., that $\delta^{13}C_{met}$ is 1 ‰ greater than the organic matter estimates for this species, which also minimizes mean bias between modeled and observed $\delta^{13}C_{oto}$ in later analyses. We consider potential drivers of isotopic enrichment of this magnitude below. If they occur, large intestinal excretory fluxes would require somewhat larger isotopic enrichments. However, an additional offset of $\delta^{13}C_{met}$ or $\Delta^{13}C_A$ of any magnitude does not affect the key physiological and environmental sensitivities (i.e., dependencies on temperature, mass, activity) that are the focus of this work.

First, we consider fractionations during otolith precipitation. The isotope effect $\Delta^{13}C_A$ includes both temperature-dependent CO_2 to bicarbonate equilibrium as well as the temperature-independent bicarbonate to aragonite equilibrium; the latter is reported as 2.7(s.d. 0.6) ‰ (range is 1.5 to 4.1 ‰ [34]). Thus an increase of $\Delta^{13}C_A$ by 0.7 ‰ as above is plausible, though greater than typical for inorganic precipitation in laboratory experiments. Because equilibrium across the DIC pool is rapid, kinetic fractionation during biological precipitation of otolith aragonite is thought to be absent or weak compared to the role of variable metabolic contributions to otoliths[40–42].

Next, we consider enrichment of metabolic carbon. Trophic enrichment on the order of 1–2 ‰ relative to the diet is expected with each step of trophic transfer in marine food webs[43–45]. Organic matter isotopic compositions reported concurrently with $\delta^{13}C_{oto}$ are usually measurements of specific tissues, often muscle. Different animal tissues vary in their isotopic enrichment or depletion by a few ‰ relative to the diet, with more protein- or mineral-rich components typically more enriched in $\delta^{13}C$ and lipid-rich components more depleted[46–48]. Because the metabolic carbon isotopic composition represents only the product of organic matter respiration transported into the internal fish fluids, selective dietary processing of carbon sources for different anabolic and catabolic needs will cause divergence of $\delta^{13}C_{met}$ from the organic matter composition of different tissues and the underlying diet. In terrestrial vertebrates, for which nearly all exhaled CO_2 is metabolically derived[49], the inferred $\delta^{13}C_{met}$ is typically within ±3 ‰ of the diet and is observed to vary systematically with species and approaches to dietary processing[50,51]. While it is not possible to isolate and directly measure only the metabolically-

derived DIC in fish fluids, these considerations suggest that a ~1 ‰ enrichment in $\delta^{13}C_{met}$ relative to reported organic matter endmembers (for Atlantic cod) is both plausible and similar to expectations given the known variability in trophic enrichment, tissue types reported, and diet utilization. Other choices to incorporate or ignore metabolic carbon enrichment relative to the underlying tissues or diet for specific analyses are discussed in *Texts S4.3, S5.4,* and *S6.1*.

Supplementary Text S3: Blood carbon partial pressures based on measurements

S3.1 Blood carbon database, normalization, and comparison to oxygen-based estimates

Experimentally determined partial pressures of CO_2 in fish blood are summarized in Table S1. This includes 24 species of Actinopterygid (ray-finned) fishes[11,52–79].

This database is meant to be illustrative rather than comprehensive, and does not include data from other clades with different blood composition that may limit the generalizability of the physiological model we present. For example, elasmobranchs have carbonic anhydrase in blood plasma or on plasma-accessible membranes which may lead to perfusion-limitation of CO_2 exchange, rather than diffusion-limitation as is typical in teleost fishes[80]. Similarly, icefish without hemoglobin appear to have plasma-accessible carbonic anhydrase, unlike other teleosts[81].

The data in Table S1 span a wide range of experimental conditions. Fish are sampled following rest or active exercise, in fresh or marine waters of widely varying P^C_w (~200–2000 µatm), and at temperatures between 1°C and 28°C. Blood is sampled from different portions of the fish circulation with higher P^C_{int} (e.g., the ventral aorta, immediately prior to ventilation at the gills) or lower P^C_{int} (e.g., the dorsal aorta, immediately following the gills). Some studies directly measure P^C_{int}, while other studies calculate this value from other carbonate system parameters. Empirical distributions of experimental P^C_{int} and P^C_w are plotted in **Fig. S2**.

A key feature of this database is that P^C_{int} always exceeds P^C_w (and 95% of P^C_{int} are at least three times greater than P^C_w). Because of the wide range of experimental conditions, for comparability of resting P^C_{int} in **Fig. 2A** we recalculate these internal pressures relative to constant conditions of 15°C and P^C_w = 370 µatm using the expression for Q(T) (*Text S2.2*). Specifically, the experimental ratio of P^C_{met} / P^C_w is calculated from measured P^C_{int} and measured or estimated P^C_w, and then multiplied by [Q(15°C) / Q(T)] · 370 µatm to normalize the experimental results to a common reference. Oxygen-derived P^C_{met} values from respirometry are already normalized to 15°C in the database from which they are derived[1], and respirometry experiments are assumed to have P^C_w already similar to 370 µatm (P^C_w is not measured during oxygen respirometry, but these experiments are often short duration and start with water well-equilibrated with the atmosphere).

Considering only resting, normalized estimates gives lower values than the raw data, but P^C_{met} nonetheless generally exceeds P^C_w, consistent with the results of the physiological model presented in this work. This contrasts with the prevailing interpretation of otolith carbon isotopic ratios that environmental carbon dominates internal carbon and resulting otolith composition (e.g., [82]), i.e., that $P^C_{met} \ll P^C_w$. This discrepancy may arise because the most commonly used

otolith endmember mixing calculation, adopted from early studies of coral carbonates (e.g., [83]), did not derive from a process-based DIC mass balance and specifically did not consider ventilatory fluxes. While metabolism generates a pool of DIC isotopically similar to the underlying organic matter, ventilation exchanges only the CO_2 fraction (*Text S1.1*). Using the isotopic endmembers dictated by the carbon mass balance halves the range between metabolic and seawater carbon compositions compared to the prior mixing model, implying that internal ^{13}C is more sensitive to metabolism than previously assumed.

S3.2 Caveats for comparison of carbon and oxygen-based estimates

Comparison of the database in Table S1 to oxygen-derived values presents some complications. Different taxonomic groups are represented in each database, and respirometry and blood chemistry measures are not collected in the same experiments preventing direct comparison under the same conditions. Further, varied and sometimes ambiguous experimental conditions lead to necessarily coarse approximations in this analysis, which limits the accuracy of the normalization approach applied above. For example, P^C_w may differ by >1500 μatm across studies, but can usually only be approximated within a few hundred μatm if not otherwise reported. Experimentally induced exercise or diet shifts could also lead to short-duration but systematic changes in respiratory stoichiometry (*Text S2.2*). Despite these potentially complicating factors, the distributions are indistinguishable (Table 2).

Moreover, caveats related to P^C_w largely do not apply to the comparison of active to resting ratios (***SMS***) estimated from each database. Paired blood carbon measurements from active and resting animals in the same experiments share similar experimental methods and conditions. The resulting distributions of ***SMS*** from each database are indistinguishable as well (Table 2), despite the different species and experimental approaches represented. These findings strengthen the case for a strong link between P^C_{met} and P^O_{met} across a range of activity levels.

Supplementary Text S4: Variable estimation for Pacific cod

S4.1. Fish data

Otolith isotopic data and fish age were previously reported from 40 Pacific cod (*Gadus macrocephalus*)[84,85]; sections between the core and edge of four representative otoliths from this dataset are plotted in **Fig. 4A**. The length and weight of each fish at the time of catch was obtained as part of the National Oceanic and Atmospheric Administration Alaska Fisheries Science Center, Resource Assessment and Conservation Engineering surveys[86]. An idealized timeseries of fish biomass is estimated by concurrently fitting Von Bertalanffy relationships[87] to the length, weight, and age data of the sampled fish:

Eq. S4.1.1 $M = \alpha_0 \cdot (L^{\alpha_1})$

Eq. S4.1.2 $L = \beta_0 \cdot (1 - e^{-\beta_1 \cdot (A - \beta_2)})$

Eq. S4.1.3 $M = \gamma_0 \cdot (1 - e^{-\beta_1 \cdot (A - \beta_2)})^{\alpha_1}$

Here M = mass, L = length, and A = age. The various coefficients (α_n, β_n, γ_n) have varied mathematical and biological interpretations in the fisheries literature but are treated here

solely as empirical parameters to generate an idealized biomass timeseries. The mass of the fish at the time of collection was never less than 85 g, but sampled otolith increments derived from post-flexion larvae and juvenile life stages at much smaller size. In order to extend the biomass timeseries to lower mass, initial larval mass was estimated as $8{\cdot}10^{-5}$ g[88] and a weighting function of the following form applied to the $\log_{10}$(M) to approximate masses between $8{\cdot}10^{-5}$ g and 85 g:

Eq. S4.1.4 $$\log_{10}(\mathrm{M}) = \rho_0 \cdot e^{-\rho_1 \cdot A} + \rho_3$$

This functional form is not grounded in theory, but was instead selected to generate a plausible curve for low mass that is broadly consistent with larval growth of other fish in the absence of well-constrained values for this species.

The enrichment of $\delta^{13}C_{oto}$ over the first year of life is a key feature of the observations. However, the absence of paired and validated measures of age, animal size, and otolith increments for animals less than a year of age necessarily limits how well model timeseries can approximate real ontogeny. A number of other factors limit the utility of a single idealized model timeseries in approximating the any particular animal's ontogeny immediately following hatching. These include the unknown relationships between early life otolith isotopic changes and: (a) the initial composition of the otolith inherited from the parent and (b) the endogenous contributions from yolk; (c) the potential for systematic hypoxia trait shifts associated with the transition from cutaneous to gill ventilation at a few weeks age; (d) the potentially large variations in growth rate between animals.

In general, the earliest age that can be distinguished in the otolith record is the first summer (~ 6 months age), which is characterized by lower $\delta^{18}O_{oto}$ characteristic of the relatively warm, summertime surface waters which the Pacific cod larvae inhabit[85]. Given the increasing potential for misalignment in the magnitude and timing of modeled and observed otolith records before this age, we restrict the model extrapolation to no less than 6 months of age. This corresponds to ~0.25 g mass, or around half of the range in orders of magnitude between the measured juvenile and larval masses referenced above.

S4.2. Hypoxia traits

Hypoxia traits have not been specifically measured for Pacific cod, so we use typical parameters from the physiological databases[1,3]. The hypoxia tolerance and temperature sensitivity are assumed to be similar to Atlantic cod—$\boldsymbol{V_h}$ = 0.05 atm (Atlantic cod is 0.062 atm), $\boldsymbol{E_o}$ = 0.35 eV with $\partial \boldsymbol{E_o}/\partial T$ = 0.01 eV/°C—but, at a reference temperature of 4°C to align with typical environmental temperatures where Pacific cod are observed. The net allometric dependence $\boldsymbol{\varepsilon}$ = -0.1 represents a typical value for organisms on the order of 1–100 g mass (Atlantic cod has $\boldsymbol{\varepsilon}$ = -0.06 at higher masses of 500-2000 g). The assumed active to resting ratio ***SMS*** = 2.5 is similar to the average of the interspecific distribution estimated from biogeography as well as the intraspecific distribution for wild Atlantic cod based on the otolith datasets in this work. Sensitivity analyses across a range of plausible values suggest that these parameters may contribute to observed variations in otolith composition between individuals (e.g., those observed in **Fig. 4A**), but are likely secondary to dietary shifts in driving the general ontogenic trend.

While it is possible to infer and optimize parameter estimates for specific records, we use the fixed parameter values above to illustrate how hypoxia traits generate plausible ontogenic records from first principles.

S.4.3. Isotopic endmembers

We approximate adult Pacific cod tissue composition using data from the Northern Gulf of Alaska, $\delta^{13}C_{om}$ ~ -17.5 ‰ (bulk composition, not the lipid-free fraction[89]). Post-flexion Pacific cod larvae are assumed to have a tissue composition similar to the composition of zooplankton and juvenile herring and pollock from the Bering Sea and Northern Gulf of Alaska[90,91], $\delta^{13}C_{om}$ ~ -22.5 ‰. In both cases, we assume that $\delta^{13}C_{met} = \delta^{13}C_{om} + 1$ ‰ as with Atlantic cod (*Text S.2.4*).

The DIC isotopic composition is approximated from available surface measurements and depth profiles in the Bering Sea and Chuchki Sea[92,93], with annual mean $\delta^{13}C_{DIC}$ ~ 1.3 ‰ in the surface where cod larvae are typically found and $\delta^{13}C_{DIC}$ ~ 0.5 ‰ at 100–150 m depth where the demersal adults are generally found. These values are used to calculate the $\delta^{13}C_w$ of $CO_{2(aq)}$ used in the physiological model. There is sparse information about the seasonality of $\delta^{13}C_{DIC}$ in this region. Therefore we use the annual mean seawater composition regardless of season for this proof-of-concept demonstration. To the extent that seasonal variations in $\delta^{13}C_w$ do influence Pacific cod otoliths, more enriched surface ocean values in summer could shift the modeled initial (post-flexion larval) $\delta^{13}C_{oto}$ to slightly more positive values (still depleted relative to adults), but should have more limited influence as the animals shift to typical adult depth ranges.

S4.4. Hydrographic timeseries

Idealized monthly timeseries of temperature, pH, and P^C_w were generated for Bering Sea conditions corresponding to larval and adult environments of Pacific cod.

Sea surface temperature in the coastal regions of the Northern Gulf of Alaska and the Bering Sea shelf has strong spatial and depth variation, but generally has a seasonal cycle of ~9°C, with minimum temperatures around March and maximum values around September[94–96]. For larval cod in the surface ocean, an idealized, stationary temperature timeseries is generated as a sine function with both annual mean and amplitude of 4.5°C, with the temperature time series beginning at the temperature minimum in March approximately when Pacific cod hatch. While adult cod experience similar timing of seasonal temperature variations, they undergo depth migrations that maintain their environmental temperature within a narrower range[97]; the idealized temperature timeseries for adults spans 3–7°C. The in situ temperatures recorded from tagged fish are notably warmer than the ~0°C bottom temperatures recorded on the bottom trawls used to collect these animals.

Bering Sea pH and pCO_2 records are limited in geographic, depth, and seasonal coverage. Therefore we apply the same carbonate system variables to all Pacific cod life stages. Compared to temperature, the seasonal maxima and minima in pH and P^C_w appear to be shifted 2–3 months earlier in the year.

Ocean pH is approximated from the climatological monthly output at 75 m depth of a regional numerical model[98,99]. This model field for the Eastern Bering Sea shelf has similar seasonal timing but lower magnitude and amplitude than the surface record at the National Oceanic and Atmospheric Administration M2 mooring farther offshore[96], as expected given the depth difference. Otolith composition is insensitive to environmental pH compared to the other parameters considered.

For P^C_w, either surface climatological observations[96,100] or 75 m depth model fields[98,99] provide similar estimates of the mean (~350–400 μatm) and seasonal timing of variations, but different seasonal amplitude (~50-100 μatm for observational climatologies versus ~200 μatm for the model climatology). The model forcing used for **Fig. 4** uses a 100 μatm seasonal amplitude. Increasing the seasonal amplitude in P^C_w to 200 μatm increases the total seasonal range of $\delta^{13}C_{oto}$ in adults (beyond the first year) by an additional 0.5‰. Thus when considering the particularly large seasonal variations in some coastal environments, P^C_w could begin to contribute to seasonal oscillations in otolith composition to a similar degree as temperature.

S4.5. Life history

Using an idealized life history of Pacific cod, we generate a logistic function characterizing the transition of diet, seawater composition, and temperature during ontogeny. Specifically, larval and small juvenile cod are assumed to spend 8 months in the surface ocean and then transition to a typical adult diet, depths, and diel vertical migration behaviors over another 6 months[97,101–103]. In other words, the idealized life history assumes that Pacific cod largely behave like adults by their second summer, and neglects some of the intermediate environmental transitions of post-settlement juveniles. The functional expression of this provides the fractional contribution of larval and adult characteristics for use in the isotopic model (e.g., the isotopic endmembers and hydrographic properties of the environment as a function of time). The goal of this parameterization is to provide smoothly varying shifts in the model otolith ontogeny that are consistent with observed life history and habitat characteristics. This approach does not represent a particular empirical or theoretical fit to observed ontogenic changes in specific animals, however more complete and concurrent observations of physiological parameters and environmental variation with time (e.g., from rearing experiments) could be used to calibrate specific parameters of interest and thus better interpret life history from the otoliths of archival or wild-caught animals.

Supplementary Text S5: Variable estimation for global analysis

S5.1. Distributions of hypoxia traits, trophic levels, and tissue isotopic composition

Empirical probability distribution functions are fit to the interspecific compilation of experimentally measured hypoxia traits ($\boldsymbol{E_o}$ and $\boldsymbol{V_h} \cdot \boldsymbol{SMS}$, with $\boldsymbol{SMS}$ estimated by species-level $\boldsymbol{\Phi_c}$ [1]), in order to describe the active P^C_{met} and its biological temperature sensitivity across all known species.

Similarly, an empirical probability distribution function is fit to a database of trophic levels across fish species[104]; the trophic level is estimated from dietary composition when available,

and the approximation from food sources otherwise). The same trophic level may be associated with different isotopic compositions because of regional variations in the isotopic composition of the phytoplankton community and the base of the food web. In particular, phytoplankton generate organic carbon during photosynthesis that is 20-30 ‰ depleted relative to surface ocean $\delta^{13}C_{DIC}$ [105]. While ecosystem modeling to account for variations in phytoplankton community structure and potential differences in photosynthetic isotope effects (e.g., for diatoms versus diazotrophs[106]) is beyond the scope of this work, variations in upper ocean $\delta^{13}C_{DIC}$ can be used to generate a first order estimate of associated geographic patterns in $\delta^{13}C_{om}$.

First, the trophic level distribution was translated into a distribution of possible enrichments of bulk tissue organic matter relative to local seawater composition ($\Delta_{om\text{-}DIC} = \delta^{13}C_{om} - \delta^{13}C_{DIC}$) using the average trophic level and isotopic enrichments from the datasets reported in Table S2. For these datasets, the mean(standard deviation) of trophic level was 4.1(0.3), and $\Delta_{TL,4.1}$ = -19(2). The estimated tissue composition for one of the cod datasets used the mean of another experiment at the same facility that was already included in Table S2, so only 15 of the 16 datasets were used for this analysis. Next, the trophic enrichment factor for any trophic level in the distribution was then estimated using this intercept and a slope of +1.5‰ per trophic level; $\delta^{13}C$ of the carbon ultimately respired by fish is elevated by 1–2 ‰ with each step of trophic transfer in marine food webs[43–45]. Thus $\Delta_{TL,i} = -19 + 1.5 \cdot (TL_i - 4.1)$, and $\delta^{13}C_{om}$ of the tissues at any geographic location can be estimated by adding this factor to $\delta^{13}C_{DIC}$.

If the value of $\delta^{13}C_{met}$ is sufficiently well constrained, the provided equations (*Text S1.2*) may be used to recalculate $\delta^{13}C_{oto}$ normalized for diet. However, for **Fig. 5** we simply plot $\delta^{13}C_{oto}$ and $\delta^{13}C_{om}$ as measured for all species given the focus on trends and distribution of results, as well as the wide range of enrichments considered. In contrast, the species-specific cod analyses were complemented by measurement and experimental constraints on the direction and magnitude of this term, and small offsets for accuracy in the magnitude of $\delta^{13}C_{oto}$ were useful for figure clarity (*Text S.2.4*) and normalizing for different diets can help illustrate the contributions of specific factors to observed $\delta^{13}C_{oto}$ (**Fig. S3**). Across many species, the value of considering differences in the metabolic carbon isotopic composition between species is best illustrated when comparing hypoxia thresholds (**Fig. 6** and *Text S.6*).

S5.2. Hydrographic variables and seawater isotopic composition

Environmental oxygen, temperature, and nutrient fields were obtained from World Ocean Atlas (version 2018) gridded climatological fields[107]. Carbonate system variables were obtained from Global Ocean Data Analysis Project, including gridded climatological fields (GLODAPv2.2016b[108]) and the isotopic composition of $\delta^{13}C_{DIC}$ from discrete sampling (GLODAPv2.2023[109,110]).

We estimate $\delta^{13}C_{DIC}$ at every coordinate using the global multiple linear regression of discrete $\delta^{13}C_{DIC}$ (‰) with temperature (T, °C), salinity (S, PSS-78), phosphate concentration ([PO_4], μmol kg^{-1}), and apparent oxygen utilization (AOU, μmol kg^{-1}) following the procedure of Eide et al.[111]:

Eq. S5.2.1 $\delta^{13}C_{DIC} = T \cdot 0.0140(0.0004) + S \cdot 0.0205(0.0021) + [PO_4] \cdot 0.3173(0.0060)$

$$-\text{AOU}\cdot 0.00820(0.00005) + 0.2061(0.0021)$$

The regression model $\delta^{13}C_{DIC}$ fits the available observations well (mean bias $2x10^{-15}$, RMSE 0.24 ‰, with n=970,187, df regression 4, df error 31,696). Additional variance in $\delta^{13}C_{DIC}$ is expected in the surface ocean such that different regions may have different empirical fits, though the global regression in Eq. S5.2.1 is used in this work; details of the construction and robustness of this approach are provided in Eide et al.[111].

Next, $\delta^{13}C_{DIC}$ is used to calculate $\delta^{13}C_w$ of CO_2 from carbonate system isotopic fractionations[33] (*Text S.2.4*), as well as $\delta^{13}C_{met}$ as described above (*Text S.5.1*). Climatological fields of select carbonate system variable concentrations and isotopic compositions are plotted in **Fig. S4**.

S5.3. Estimates of otolith isotopic compositions

The otolith composition is then calculated for each combination of hypoxia traits, trophic level, and local hydrographic conditions using Equation 2. These combinations are weighted by the measured global distributions trophic levels and hypoxia traits—excluding those for which metabolic needs cannot be supported locally ($P^O_{met} \cdot \boldsymbol{SMS} > P^O_w$)— in order to calculate a distribution of possible otolith compositions for each location within the World Ocean Atlas gridding. This approximates unknown fish communities by assuming that these multi-parameter distributions are broadly similar at the local and global scale, other than the metabolic selection just described, and that hydrography-driven local variations in temperature and $\delta^{13}C_w$ dominate differences in the regional expression of $\delta^{13}C_{oto}$. Model output for the surface waters of a Pacific transect are presented in **Fig. S5**.

For plotting, the model distributions for each location falling within the same depth bin (**Fig. 5A**) or joint temperature and isotopic composition bin (**Fig. 5B**) are weighted together according to the fraction of total evaluated hypoxia trait combinations ('ecophysiotypes' representing the full range of expected species diversity) present in each hydrographic cell. Thus when averaging the otolith composition distribution of two water parcels, one that can support 100% and another 50% of ecophysiotypes, the two distributions are weighted by a ratio of 1:0.5 in generating the combined distribution with depth or temperature (this approach is extended to any arbitrary number of water parcels being averaged).

Because species-specific hypoxia traits and life-histories as well as community composition and trophic structure vary across marine ecosystems, individual species and ecosystems will diverge from the broad expectations presented in **Fig. 5**. Species-specific predictions can also be generated using the model and methods described in this work, as demonstrated in **Figs. 3**, **4**, and **6**.

S5.4. Otolith observations

Measured $\delta^{13}C_{oto}$ from 16 datasets and 13 species are included in Table S2, encompassing a wide range of environments and life-histories[36,38,39,84,112–120]. For comparability across species and to limit artifacts from ontogenic dietary shifts, the intra-otolith transects of $\delta^{13}C_{oto}$ in two studies are averaged to generate a mean composition (see notes in Table S2). Ancillary information like

temperature, body mass, and isotopic composition of environmental and tissue carbon is reported with some of these data, and otherwise estimated from other literature cited in Table S2. In general, sufficient data is presented so that the environmental and isotopic parameters of Equation 1 can be fully described, and compositions could be recalculated to normalize across different environmental conditions if desired. Empirical distributions of measured or estimated $\delta^{13}C_{oto}$, $\delta^{13}C_{w}$ ($CO_{2(aq)}$), and $\delta^{13}C_{om}$ from Table S2 are plotted in **Fig. S2**.

In the absence of a community repository for quality-controlled otolith isotopic measurements and metadata, this database is meant to be illustrative of interspecific trends rather than a comprehensive survey of prior work. Thus it excludes some datasets for which ancillary information is missing or difficult to estimate, prior compilations with few data per species or with reported ancillary information that is inconsistent with the underlying references, and several additional Atlantic cod datasets which are qualitatively similar to those considered in this work.

Supplementary Text S6: Estimation of hypoxia traits from isotopic measurements

S6.1. Calculation

The ratio of P^{C}_{met} / P^{C}_{w} can be calculated from Equation S1.2.9 using measured or estimated isotopic compositions and the fractionations described in *Text S2*. With measured or assumed P^{C}_{w}, P^{C}_{met} can then be related to various hypoxia traits. Using Equation S1.2.6, we calculate P^{O}_{met} and the equivalent environmental hypoxia threshold P^{O}_{w} for the observed temperature. This can be compared directly to environmental thresholds at the same temperature generated from other methods (e.g., **Fig. 6**), or normalized to a reference condition for comparison across species using Equation S1.2.3 and estimates of $\boldsymbol{E_o}$ and $\boldsymbol{\varepsilon}$; in the latter case, this estimate is defined as the active hypoxia vulnerability, $\boldsymbol{V_h} \cdot \boldsymbol{SMS}$. For the Atlantic cod case study of interspecific variation in otolith composition, we used the same starting point but assumed respirometry-derived literature values of $\boldsymbol{E_o}$ and $\boldsymbol{V_h}$ to solve for the value of $\boldsymbol{SMS}$ associated with each wild and reared cod datum. Joint probability bounds for multiple hypoxia traits could be determined concurrently from sufficiently large and precise datasets of otolith and ancillary isotopic and environmental measurements.

We made several choices to improve comparability of otolith and oxygen-based estimates of hypoxia thresholds. First, we estimated $\delta^{13}C_{met}$ from $\delta^{13}C_{om}$ differently depending on the data source (Table S2): For Atlantic cod, $\delta^{13}C_{met} = \delta^{13}C_{om} + 1$ ‰ as discussed previously. For other species with measured tissue isotopic compositions, $\delta^{13}C_{met} = \delta^{13}C_{om}$. For species without tissue measurements, but for which the isotopic composition of their diet or prey has been measured, $\delta^{13}C_{met} = \delta^{13}C_{om} + 1.5$ ‰ as was assumed when generating modeled global distributions of $\delta^{13}C_{om}$ (Text S5). Species-specific values of $\delta^{13}C_{w}$ were estimated for each dataset as described in the notes of Table S2; while local or regional datasets were used here, substituting the modeled $\delta^{13}C_{w}$ resulting from $\delta^{13}C_{DIC}$ calculated from Eq. S5.2.1 also works well. The P^{C}_{w} was estimated from ancillary information provided with the datasets and other literature values or oceanographic correlations (see notes in Table S3). Resulting otolith-derived means and ranges of P^{C}_{met} / P^{C}_{w} and P^{O}_{w} are reported in Table S3 (after filtering out unconstrained estimates, see *Text S6.2*).

Oxygen-derived P^O_w were generated either from direct respirometry[1] (when considering reared, resting Atlantic cod and red drum) or more commonly from biogeographic distributions of species. Specifically, we fit Equation 1.2.3 to global species presence data binned with respect to pO_2 and temperature following previously published methods to determine the lowest inhabited pO_2 at a given temperature and generate associated $\boldsymbol{V_h} \cdot \boldsymbol{SMS}$ and $\boldsymbol{E_o}$ (Table S3; see [121,122] for a detailed explanation of the methods). The P^O_w (at the reported in situ temperatures) was rescaled from the biogeographically-derived $\boldsymbol{V_h} \cdot \boldsymbol{SMS}$ (at 15°C) using the paired $\boldsymbol{E_o}$. These empirically fit hypoxia traits differ slightly from the respirometry-informed values for the same species, reflecting the role of ***SMS***[122], and instead provide independent estimates from those used in prior analyses. The rationale for using the biogeographically-fit traits is that the result is similar to directly taking the lowest few percent of pO_2 for fish observations at a given temperature as a threshold estimate (the ideal comparison), but represents some minimal smoothing across temperatures to account for the patchy observational distributions at any single temperature. While the respirometry-informed estimates are preferred in the preceding cod-specific analyses, substituting the biogeography-derived traits in the Atlantic and Pacific cod analyses does not qualitatively change any of the preceding findings.

S6.2. Sources of error

In practice, P^C_{met} / P^C_w becomes increasingly uncertain as the ratio becomes larger because the denominator $\delta^{13}C_{int} - \delta^{13}C_{met}$ approaches zero. Replicate uncertainties in the underlying measurements of $\delta^{13}C_{oto}$, $\delta^{13}C_{om}$, and $\delta^{13}C_{DIC}$ are on the order of 0.6, 0.3 and 0.06 ‰, respectively, in the cod datasets used in this work. We illustrate the consequences of these uncertainties and limits on the robust estimation of the P^C_{met} / P^C_w ratio using a Monte Carlo simulation of 100,000 draws of isotopic compositions including normally distributed random errors corresponding to the replicate uncertainties for the underlying measurement parameters. These define an idealized vector of P^C_{met} / P^C_w, with fixed $\delta^{13}C_{met}$ = -20‰ and $\delta^{13}C_w$ in equilibrium with $\delta^{13}C_{DIC}$ = 0‰ at 15°C, and the increasing vector of $\delta^{13}C_{int}$ required to generate that ratio. The defined ratio, the simulated ratio including measurement error, and the associated interquartile uncertainty bounds are plotted in **Fig. S6**.

Within 2-se of $\delta^{13}C_{int} - \delta^{13}C_{met} = 0$, random errors of small magnitude in the denominator produce errors in the ratio that are inversely proportional, and thus increasingly large. Within 1-se of zero, the interquartile uncertainty bounds become effectively unconstrained (100% of the defined ratio or larger), corresponding to hypoxia threshold bounds spanning anoxia to greater than the highest supersaturation of oxygen typically observed in the ocean. In other words, beyond that point fewer than 50% of results fall within the physically possible range, and qualitatively useful results are not expected given measurement uncertainties. Moreover, small random biases increasingly generate erroneous negative values ($\delta^{13}C_{int} < \delta^{13}C_{met}$) near zero. This generates negative ratios of very large magnitude, which causes an increasingly large negative, systematic bias in the mean estimate compared to the true value. Systematic biases are also likely when isotopic endmembers are indirectly estimated (e.g., from diet compositions or correlations of climatological environmental variables), which can further limit the useful range of isotopic compositions from which the metabolic carbon contribution can be estimated.

Based on this analysis, we exclude samples with calculated $\delta^{13}C_{int}$ - $\delta^{13}C_{met}$ < 0.7‰ (combined 1-se of this difference based on replicate precision) from the two analyses that rely on inversion of isotopic ratios to calculate P^{C}_{met} / P^{C}_{w} and subsequent hypoxia traits. This excludes three wild cod samples from the estimates of ***SMS*** (**Fig. 3C**) and a handful of observations from several other datasets in the calculation of environmental hypoxia thresholds (**Fig. 6**). On inspection, most of samples filtered in this manner led to P^{C}_{met} / P^{C}_{w} estimates that were well beyond the magnitude of direct blood carbon observations (with both positive and negative sign, as expected from the error analysis). This may suggest a limitation in the use of otoliths to derive hypoxia traits in organisms with unusually high internal metabolic carbon burden. For example, the marlin otolith observations included in Table S3 were not suitable for use in **Fig. 6** based on this criteria, though other factors complicated analysis of these data (reported otolith values were binned across large regions, and tissue composition was approximated from a different study and region; [118,123]). Similarly, tuna are observed to have remarkably negative $\delta^{13}C_{oto}$[124], which would likely correspond to near-zero $\delta^{13}C_{int} - \delta^{13}C_{met}$.

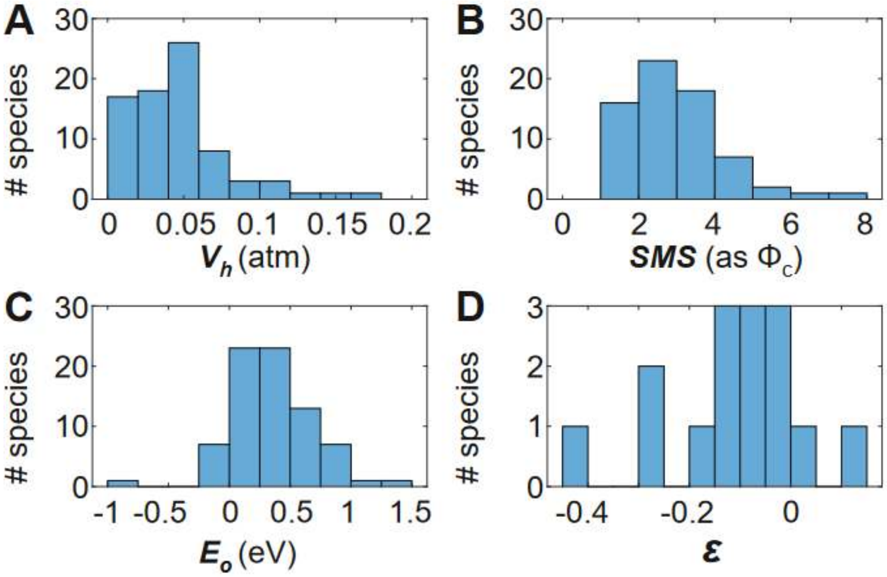

Fig. S1.

Empirical distributions of hypoxia traits from Deutsch et al.[1] (**A-C**) and Deutsch et al.[3] (**D**). (**A**) Hypoxia vulnerability, $\boldsymbol{V_h}$ at 15°C (**B**) sustained metabolic scope, ***SMS*** estimated as the species-level average from biogeographic distributions, $\boldsymbol{\Phi_c}$, (**C**) the temperature scaling of hypoxia vulnerability, $\boldsymbol{E_o}$, and (**D**) the mass scaling of hypoxia vulnerability, $\boldsymbol{\varepsilon}$.

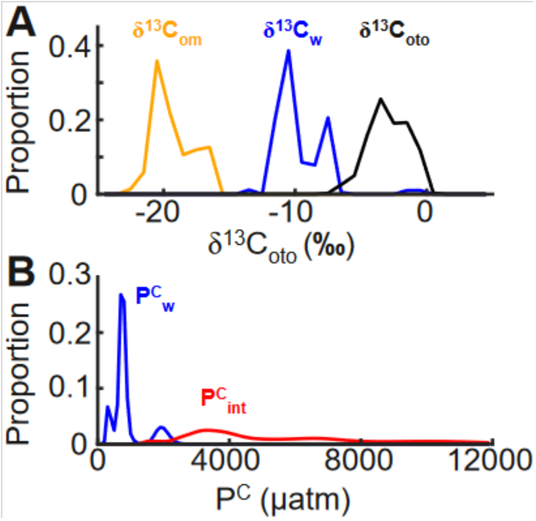

Fig. S2.

Distributions of measured or estimated (**A**) isotopic values from Table S2 and (**B**) partial pressures of $CO_{2(aq)}$ from Table S1, without normalization. All distributions are empirically smoothed using a kernel width of 10% the plotted range.

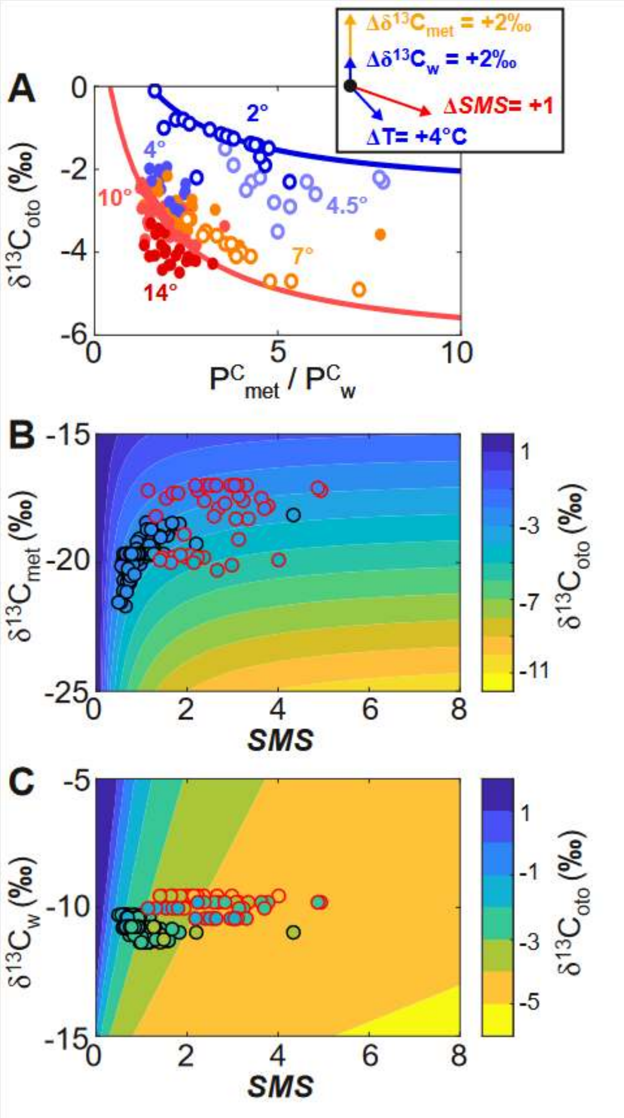

Fig. S3.

(**A**) Measured otolith isotopic composition ($\delta^{13}C_{oto}$) and inferred internal fluid carbon contributions from metabolism and seawater (P^C_{met}/P^C_w). Individual Atlantic cod (*Gadus morhua*) are grouped by environmental or experimental temperature (colors) and wild (open circles[36,39]) versus reared animals (filled circles[38,39]). Wild cod span a range of isotopic compositions for metabolic ($\delta^{13}C_{met}$) and seawater ($\delta^{13}C_w$) carbon, while the reared animals share similar isotopic endmembers. The colored curves represent model solutions with constant temperature and endmember compositions: blue is wild cod at 2°C, red is reared cod at 10°C. The boxed vector diagram represents modeled $\delta^{13}C_{oto}$ changes with environmental or metabolic shifts, relative to an initial $P^C_{met}/P^C_w = 3$ and $\delta^{13}C_{oto}$ = -3‰ (the effect sizes depend on initial conditions). (**B**) The $\delta^{13}C_{oto}$ (filled colored contours) resulting from the ranges of ***SMS*** and $\delta^{13}C_{met}$ (representative of diet and tissue compositions spanning phytoplankton to top predators), at a fixed temperature of 15°C and $\delta^{13}C_w$ = -10‰ (in equilibrium with $\delta^{13}C_{DIC}$ = 0‰, and with V_h = 0.05 atm; higher V_h shifts contours to the left). Black circles represent reared cod data, and red circles the wild cod data. The circle fill color is the $\delta^{13}C_{oto}$ recalculated at the same fixed temperature and $\delta^{13}C_w$ as the model contours (by solving for P^C_{met}/P^C_w and then recalculating the otolith composition expected from that under new conditions). (**C**) A similar model and recalculated observational $\delta^{13}C_{oto}$, but with $\delta^{13}C_w$ spanning ocean conditions and fixed $\delta^{13}C_{met}$ = -20‰. The subset of wild data with $\delta^{13}C_{met}$ farthest from -20‰ diverge moderately from the model expectations for $\delta^{13}C_{oto}$ when recalculated to the new reference condition, which may suggest variable fractionation between $\delta^{13}C_{met}$ and varied tissue compositions (*Text S2.4*). At typical ***SMS*** between 1.5 and 4, $\delta^{13}C_{oto}$ is sensitive to $\delta^{13}C_{met}$ and insensitive to $\delta^{13}C_w$.

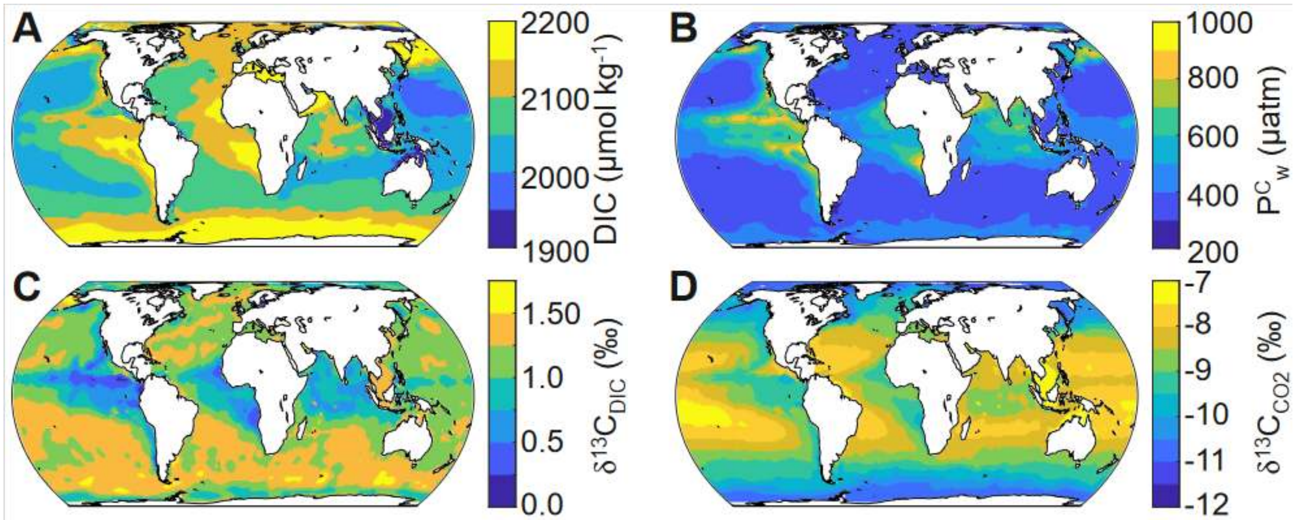

Fig. S4.

Climatological surface ocean (0–200 m average) distributions of (**A**) dissolved inorganic carbon (DIC) and (**B**) partial pressures of CO_2 ($P^C{}_w$), and associated isotopic compositions (**C**) $\delta^{13}C_{DIC}$ and (**D**) $\delta^{13}C_{CO2(aq)}$ (see *Text S5.2*). The longitude, latitude, and depth specific variable estimates are used in the global modeling analysis.

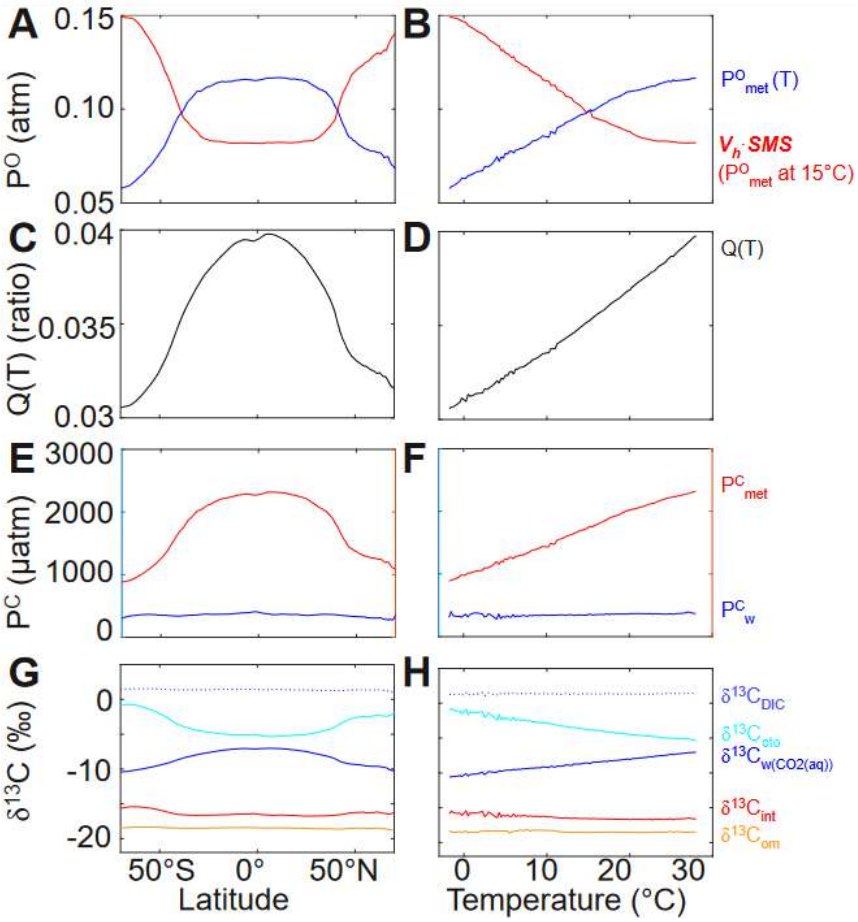

Fig. S5.

Latitudinal transects of select model outputs through the surface ocean (0 m) at 170°E in the Pacific Ocean. Outputs represent the weighted mean of hypoxia traits and diets at each coordinate. Variables are plotted against latitude in the left-hand column (panels **A**, **C**, **E**, and **G**) and against temperature in the right-hand column (panels **B**, **D**, **F**, and **H**). Variables are defined in the main and *SM* texts, as well as in the glossary (Table S4).

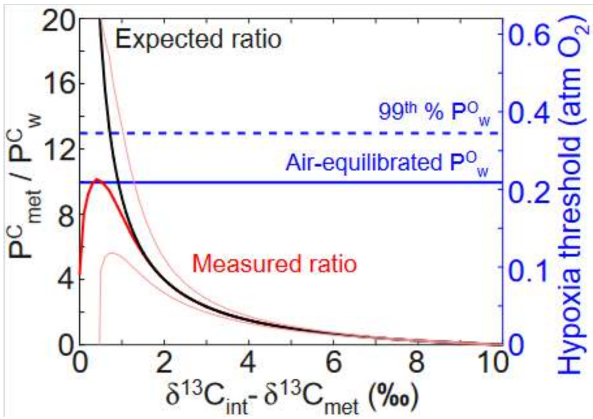

Fig. S6.

Expected errors in the estimation of P^C_{met}/P^C_w (and resulting hypoxia thresholds in the environment) from isotopic measurements with analytical uncertainties, as a function of the difference between the internal and metabolic carbon compositions. The thick black line represents the expected ratio imposed by the parameters described in *Text S6.2*. The thick red line represents the mean estimate of the ratio measured across a Monte Carlo simulation with randomly distributed analytical errors, and the thin red lines are the interquartile range of simulated results. The solid blue line is the P^O_w at equilibrium with atmospheric pO_2, which most of the surface ocean is expected to be near, and the dashed blue line represents the 99th percentile of climatological P^O_w in the ocean, beyond which higher hypoxia thresholds (and equivalent P^C_{met}/P^C_w) are ecologically implausible. See *Text S6.2* for additional details.

Table S1. Measured and inferred internal carbon pools of Actinopterygid fish (external spreadsheet).

Table S2. Otolith isotopic data and metadata used in analyses (external spreadsheet).

Table S4. Extended glossary of parameters used in the *Supplementary Materials*.

Parameter	Units	Definition	Estimation procedure
Isotopic parameters			
α	non-dim.	Equilibrium fractionation factor, ratio of R values	Calculated from measured R
δ	‰	Normalized isotopic ratio, $\delta = (R / R_{standard} - 1) \cdot 1000$	Calculated from measured R
Δ	‰	Isotopic difference	Calculated from α (as isotope effect between two chemical species) or as difference in δ between a measured condition and a reference state
R	non-dim.	Isotopic ratio, e.g., $^{13}C/^{12}C$	Mass spectrometry
Partial pressures			
P^C & P^O	atm	Partial pressure of CO_2 & O_2	Measured or calculated from carbonate system equilibria and hydrography
Rates and associated variables			
$\alpha^C{}_D$ & $\alpha^O{}_D$	mol C (O_2) g^{-1} h^{-1}	Respiratory rate of DIC (O_2) production per unit biomass	Calculated from O_2 rate and respiratory quotient
$\alpha^C{}_S$ & $\alpha^O{}_S$	mol C (O_2) g^{-1} atm^{-1} h^{-1}	Ventilatory rate coefficient for CO_2 & O_2, per unit biomass	Calculated from O_2 rate and ventilatory quotient
$D_C(T)$ & $D_O(T)$	m^2 hr^{-1}	Diffusion coefficient of CO_2 or O_2	Calculated from empirical or theoretical diffusivity parameterizations
$K_C(T)$ & $K_O(T)$	mol m^{-3} atm^{-1}	Henry's solubility coefficient, K_H, for CO_2^* (H_2CO_3+$CO_{2(aq)}$) & O_2	Calculated from empirical solubility parameterizations
Q(T)	non-dim.	The temperature-dependent stoichiometric coefficient	Calculated the ratio R_Q/V_Q
R_Q	non-dim.	Respiratory quotient, the ratio of $\boldsymbol{\alpha^C{}_D/\alpha^O{}_D}$	Measured during O_2 and CO_2 respirometry
V_Q	non-dim.	Ventilatory quotient, the ratio of $\boldsymbol{\alpha^C{}_S/\alpha^O{}_S}$	Calculated based on the relative diffusivity across the gill
Hypoxia traits and associated variables			
B	non-dim.	Body mass normalized to reference mass	Measured or estimated from length-mass relationships
$\boldsymbol{\varepsilon}$	non-dim.	Allometric scaling exponent of supply to demand	Fit to $P^O{}_{met}$ during O_2 respirometry across a range of body masses
$\boldsymbol{E_o}$	eV	Temperature scaling of demand to supply	Fit to $P^O{}_{met}$ during O_2 respirometry across a range of temperatures
k_B	eV K^{-1}	Boltzmann constant	Physical constant
SMS	non-dim.	Sustained metabolic scope, the ratio of sustained to resting metabolic rates	Estimated experimentally, or from biogeographic thresholds of animal observations (as $\boldsymbol{\Phi_c}$)
T'	K^{-1}	Inverse temperature, $T' = 1/T - 1/T_{ref}$ in K	Calculated relative to reference temperature
$\boldsymbol{V_h}$	Atm	Hypoxia vulnerability at a reference temperature and mass	Experimentally determined $P^O{}_{met}$ during O_2 respirometry at reference conditions

Supplemental references

1. Deutsch, C., Penn, J. L. & Seibel, B. Metabolic trait diversity shapes marine biogeography. *Nature* **585**, 557–562 (2020).
2. Deutsch, C., Ferrel, A., Seibel, B., Pörtner, H.-O. & Huey, R. B. Climate change tightens a metabolic constraint on marine habitats. *Science (1979)* **348**, 1132–1135 (2015).
3. Deutsch, C. *et al.* Impact of warming on aquatic body sizes explained by metabolic scaling from microbes to macrofauna. *Proceedings of the National Academy of Sciences* **119**, e2201345119 (2022).
4. Solomon, C. T. *et al.* Experimental determination of the sources of otolith carbon and associated isotopic fractionation. *Canadian Journal of Fisheries and Aquatic Sciences* **63**, 79–89 (2006).
5. Caut, S., Angulo, E. & Courchamp, F. Variation in discrimination factors (Δ^{15} N and Δ^{13} C): the effect of diet isotopic values and applications for diet reconstruction. *Journal of Applied Ecology* **46**, 443–453 (2009).
6. Whittamore, J. M., Cooper, C. A. & Wilson, R. W. HCO3− secretion and CaCO3 precipitation play major roles in intestinal water absorption in marine teleost fish in vivo. *American Journal of Physiology-Regulatory, Integrative and Comparative Physiology* **298**, R877–R886 (2010).
7. Hunn, J. B. Blood chemistry values for some fishes of the upper Mississippi River. *J Minn Acad Sci* **38**, 19–21 (1972).
8. Wood, S., Johansen, K. & Weber, R. Effects of ambient PO2 on hemoglobin-oxygen affinity and red cell ATP concentrations in a benthic fish, pleuronectes platessa. *Respir Physiol* **25**, 259–267 (1975).
9. Gilmour, K. & Perry, S. The effects of hypoxia, hyperoxia or hypercapnia on the acid-base disequilibrium in the arterial blood of rainbow trout. *Journal of Experimental Biology* **192**, 269–284 (1994).
10. Eddy, F. B., Lomholt, J. P., Weber, R. E. & Johansen, K. Blood respiratory properties of rainbow trout (Salmo gairdneri) kept in water of high CO2 tension. *Journal of Experimental Biology* **67**, 37–47 (1977).
11. Korsmeyer, K. E., Lai, N. C., Shadwick, R. E. & Graham, J. B. Oxygen transport and cardiovascular responses to exercise in the yellowfin tuna Thunnus albacares. *Journal of Experimental Biology* **200**, 1987–1997 (1997).
12. Soncini, R. & Glass, M. L. The effects of temperature and hyperoxia on arterial PO2 and acid-base status in Piaractus mesopotamicus. *J Fish Biol* **51**, 225–233 (1997).
13. Kutty, M. N. Respiratory quotients in goldfish and rainbow trout. *Journal of the Fisheries Research Board of Canada* **25**, 1689–1728 (1968).
14. Wang, S., Carter, C. G., Fitzgibbon, Q. P. & Smith, G. G. Respiratory quotient and the stoichiometric approach to investigating metabolic energy substrate use in aquatic ectotherms. *Rev Aquac* **13**, 1255–1284 (2021).
15. Hamme, R. Ocean gas solubility and diffusion functions for MATLAB. *Script* Preprint at https://web.uvic.ca/~rhamme/download.html (2013).
16. Ferrell, R. T. & Himmelblau, D. M. Diffusion coefficients of nitrogen and oxygen in water. *J Chem Eng Data* **12**, 111–115 (1967).
17. Garcia H & Gordon L. Erratum: Oxygen solubility in seawater: Better fitting equations. *Limnol Oceanogr* **38**, 643–656 (1993).

18. Garcia, H. E. & Gordon, L. I. Oxygen solubility in seawater: Better fitting equations. *Limnol Oceanogr* **37**, 1307–1312 (1992).
19. Weiss, R. F. Carbon dioxide in water and seawater: the solubility of a non-ideal gas. *Mar Chem* **2**, 203–215 (1974).
20. Dickson, A., Sabine, C. & Christian, J. (eds.). *Guide to Best Practices for Ocean CO2 Measurement*. vol. PICES Spec. Pub. 3 (North Pacific Marine Science Organization, Sidney, British Columbia, 2007).
21. Zeebe, R. E. On the molecular diffusion coefficients of dissolved CO2,HCO3-, and CO32- and their dependence on isotopic mass. *Geochim Cosmochim Acta* **75**, 2483–2498 (2011).
22. Millero, F. J. Carbonate constants for estuarine waters. *Mar Freshw Res* **61**, 139–142 (2010).
23. Boutilier, R. G., Heming, T. A. & Iwama, G. K. Gills: Anatomy, gas transfer, and acid-base regulation. Appendix: Physicochemical parameters for use in fish respiratory physiology. in *Fish Physiology* (eds. Hoar, W. S. & Randall, D. J.) vol. 10 403–430 (Academic Press, 1984).
24. Boutilier, R. G., Iwama, G. K., Heming, T. A. & Randall, D. J. The apparent pK of carbonic acid in rainbow trout blood plasma between 5 and 15°C. *Respir Physiol* **61**, 237–254 (1985).
25. Becker, A. G. *et al.* Plasma ion levels of freshwater and marine/estuarine teleosts from Southern Brazil. *Neotropical Ichthyology* **9**, 895–900 (2011).
26. Kültz, D. Physiological mechanisms used by fish to cope with salinity stress. *Journal of Experimental Biology* **218**, 1907–1914 (2015).
27. Lewis, E., Wallace, D. & Allison, L. *Program Developed for CO2 System Calculations*. vol. ORNL/CDIAC-105 (Oak Ridge National Laboratory, Oak Ridge, Tennessee, 1998).
28. Dickson, A. G. pH scales and proton-transfer reactions in saline media such as sea water. *Geochim Cosmochim Acta* **48**, 2299–2308 (1984).
29. Hunter, K. A. The temperature dependence of pH in surface seawater. *Deep Sea Research Part I: Oceanographic Research Papers* **45**, 1919–1930 (1998).
30. Cameron, J. N. Regulation of blood pH in teleost fish. *Respir Physiol* **33**, 129–144 (1978).
31. Millero, F. J. Thermodynamics of the carbon dioxide system in the oceans. *Geochim Cosmochim Acta* **59**, 661–677 (1995).
32. Zeebe, R. & Wolf-Gladrow, D. *CO2 in Seawater: Equilibrium, Kinetics, Isotopes*. vol. 65 (Elsevier Oceanography Series, 2001).
33. Zhang, J., Quay, P. D. & Wilbur, D. O. Carbon isotope fractionation during gas-water exchange and dissolution of CO2. *Geochim Cosmochim Acta* **59**, 107–114 (1995).
34. Romanek, C. S., Grossman, E. L. & Morse, J. W. Carbon isotopic fractionation in synthetic aragonite and calcite: Effects of temperature and precipitation rate. *Geochim Cosmochim Acta* **56**, 419–430 (1992).
35. Morel, F. & Hering, J. *Principles and Applications of Aquatic Chemistry*. (Wiley, New York, New York, 1993).
36. Jamieson, R. Environmental history of northern cod from otolith isotopic analysis. (McMaster University, Hamilton, Ontario, 2001).
37. Jamieson, R. E., Schwarcz, H. P. & Brattey, J. Carbon isotopic records from the otoliths of Atlantic cod (Gadus morhua) from eastern Newfoundland, Canada. *Fish Res* **68**, 83–97 (2004).

38. Chung, M.-T., Trueman, C. N., Godiksen, J. A., Holmstrup, M. E. & Grønkjær, P. Field metabolic rates of teleost fishes are recorded in otolith carbonate. *Commun Biol* **2**, 24 (2019).
39. Gao, Y., Schwarcz, H. P., Brand, U. & Moksness, E. Seasonal Stable Isotope Records of Otoliths from Ocean-pen Reared and Wild Cod, Gadus morhua. *Environ Biol Fishes* **61**, 445–453 (2001).
40. Kalish, J. 13C and 18O isotopic disequilibria in fish otoliths: metabolic and kinetic effects. *Marine Ecology Progress Series* **75**, 191–203 (1991).
41. Thorrold, S. R., Campana, S. E., Jones, C. M. & Swart, P. K. Factors determining δ13C and δ18O fractionation in aragonitic otoliths of marine fish. *Geochim Cosmochim Acta* **61**, 2909–2919 (1997).
42. Høie, H., Folkvord, A. & Otterlei, E. Effect of somatic and otolith growth rate on stable isotopic composition of early juvenile cod (Gadus morhua L) otoliths. *J Exp Mar Biol Ecol* **289**, 41–58 (2003).
43. Rau, G. H. *et al.* Animal C/C Correlates with Trophic Level in Pelagic Food Webs. *Ecology* **64**, 1314–1318 (1983).
44. Sholto-Douglas, A., Field, J., James, A. & van der Merwe, N. 13C/12 C and 15N/14 N isotope ratios in the Southern Benguela Ecosystem: indicators of food web relationships among different size-classes of plankton and pelagic fish; differences between fish muscle and bone collagen tissues. *Marine Ecology Progress Series* **78**, 23–31 (1991).
45. Cherel, Y. *et al.* Isotopic niches of fishes in coastal, neritic and oceanic waters off Adélie land, Antarctica. *Polar Sci* **5**, 286–297 (2011).
46. Tieszen, L. L. & Fagre, T. Effect of diet quality and composition on the isotopic composition of respiratory CO2, bone collagen, bioapatite, and soft tissues. in *Prehistoric Human Bone* 121–155 (Springer Berlin Heidelberg, Berlin, Heidelberg, 1993). doi:10.1007/978-3-662-02894-0_5.
47. Pinnegar, J. K. & Polunin, N. V. C. Differential fractionation of δ13C and δ15N among fish tissues: implications for the study of trophic interactions. *Funct Ecol* **13**, 225–231 (1999).
48. Li, C.-H., Roth, J. D. & Detwiler, J. T. Isotopic turnover rates and diet-tissue discrimination depend on feeding habits of freshwater snails. *PLoS One* **13**, e0199713 (2018).
49. McConnaughey, T. A., Burdett, J., Whelan, J. F. & Paull, C. K. Carbon isotopes in biological carbonates: Respiration and photosynthesis. *Geochim Cosmochim Acta* **61**, 611–622 (1997).
50. Passey, B. H. *et al.* Carbon isotope fractionation between diet, breath CO2, and bioapatite in different mammals. *J Archaeol Sci* **32**, 1459–1470 (2005).
51. Cerling, T. E. *et al.* CH4/CO2 ratios and carbon isotope enrichment between diet and breath in herbivorous mammals. *Front Ecol Evol* **9**, (2021).
52. Currie, S. & Tufts, B. L. An analysis of carbon dioxide transport in arterial and venous blood of the rainbow trout, Oncorhynchus mykiss, following exhaustive exercise. *Fish Physiol Biochem* **12**, 183–192 (1993).
53. Holeton, G. F., Neumann, P. & Heisler, N. Branchial ion exchange and acid-base regulation after strenuous exercise in rainbow trout (Salmo gairdneri). *Respir Physiol* **51**, 303–318 (1983).

54. Thomas, S. Changes in blood acid-base balance in trout (Salmo gairdneri Richardson) following exposure to combined hypoxia and hypercapnia. *J Comp Physiol* **152**, 53–57 (1983).
55. Toews, D. P., Holeton, G. F. & Heisler, N. Regulation of the acid-base status during environmental hypercapnia in the marine teleost fish Conger conger. *Journal of Experimental Biology* **107**, 9–20 (1983).
56. Turner, J. D., Wood, C. M. & Höbe, H. Physiological consequences of severe exercise in the inactive benthic flathead sole (Hippoglossoides elassodon): A comparison with The active pelagic rainbow trout (Salmo gairdneri). *Journal of Experimental Biology* **104**, 269–288 (1983).
57. van den Thillart, G., Randall, D. & Hoa-Ren, L. CO2 and H+ excretion by swimming coho salmon, Oncorhynchus kisutch. *Journal of Experimental Biology* **107**, 169–180 (1983).
58. Perry, S. F., Daxboeck, C., Emmett, B., Hochachka, P. W. & Brill, R. W. Effects of temperature change on acid-base regulation in skipjack tuna (Katsuwonus pelamis) blood. *Comp Biochem Physiol A Physiol* **81**, 49–53 (1985).
59. Schwalme, K. & Mackay, W. C. The influence of exercise–handling stress on blood lactate, acid–base, and plasma glucose status of northern pike (Esox lucius L.). *Can J Zool* **63**, 1125–1129 (1985).
60. Milligan, C. L. & Wood, C. M. Regulation of blood oxygen transport and red cell pHi after exhaustive activity in rainbow trout (Salmo gairdneri) and starry flounder (Platichthys stellatus). *Journal of Experimental Biology* **133**, 263–282 (1987).
61. Thomas, S., Poupin, J., Lykkeboe, G. & Johansen, K. Effects of graded exercise on blood gas tensions and acid-base characteristics of rainbow trout. *Respir Physiol* **68**, 85–97 (1987).
62. Tang, Y., Lin, H. & Randall, D. J. Compartmental distributions of carbon dioxide and ammonia in rainbow trout at rest and following exercise, and the effect of bicarbonate infusion. *Journal of Experimental Biology* **169**, 235–249 (1992).
63. Nelson, J., Tang, Y. & Boutilier, R. The effects of salinity change on the exercise performance of two Atlantic cod (Gadus morhua) populations inhabiting different environments. *Journal of Experimental Biology* **199**, 1295–1309 (1996).
64. Egginton, S. A comparison of the response to induced exercise in red- and white-blooded Antarctic fishes. *Journal of Comparative Physiology B* **167**, 129–134 (1997).
65. Korsmeyer, K. E., Lai, N. C., Shadwick, R. E. & Graham, J. B. Heart rate and stroke volume contribution to cardiac output in swimming yellowfin tuna: response to exercise and temperature. *Journal of Experimental Biology* **200**, 1975–1986 (1997).
66. Gonzalez, R. J., Milligan, L., Pagnotta, A. & McDonald, D. G. Effect of air breathing on acid-base and ion regulation after exhaustive exercise and during Low pH exposure in the bowfin, Amia calva. *Physiological and Biochemical Zoology* **74**, 502–509 (2001).
67. McKenzie, D., Taylor, E., Dalla Valle, A. & Steffensen, J. Tolerance of acute hypercapnic acidosis by the European eel (Anguilla anguilla). *Journal of Comparative Physiology B* **172**, 339–346 (2002).
68. Larsen, B. K., Pörtner, H.-O. & Jensen, F. B. Extra- and intracellular acid-base balance and ionic regulation in cod (Gadus morhua) during combined and isolated exposures to hypercapnia and copper. *Mar Biol* **128**, 337–346 (1997).

69. Lee, K.-S., Kita, J. & Ishimatsu, A. Effects of lethal levels of environmental hypercapnia on cardiovascular and blood-gas status in yellowtail, Seriola quinqueradiata. *Zoolog Sci* **20**, 417–422 (2003).
70. Bernier, N. J., Brauner, C. J., Heath, J. W. & Randall, D. J. Oxygen and carbon dioxide transport during sustained exercise in diploid and triploid chinook salmon (Oncorhynchus tshawytscha). *Canadian Journal of Fisheries and Aquatic Sciences* **61**, 1797–1805 (2004).
71. Hayashi, M., Kita, J. & Ishimatsu, A. Acid-base responses to lethal aquatic hypercapnia in three marine fishes. *Mar Biol* **144**, 153–160 (2004).
72. Michaelidis, B., Spring, A. & Pörtner, H. O. Effects of long-term acclimation to environmental hypercapnia on extracellular acid–base status and metabolic capacity in Mediterranean fish Sparus aurata. *Mar Biol* **150**, 1417–1429 (2007).
73. Roth, B. & Rotabakk, B. T. Stress associated with commercial longlining and recreational fishing of saithe (Pollachius virens) and the subsequent effect on blood gases and chemistry. *Fish Res* **115–116**, 110–114 (2012).
74. Harter, T. S. *et al.* Preferential intracellular pH regulation represents a general pattern of pH homeostasis during acid–base disturbances in the armoured catfish, Pterygoplichthys pardalis. *Journal of Comparative Physiology B* **184**, 709–718 (2014).
75. Ern, R. & Esbaugh, A. J. Hyperventilation and blood acid–base balance in hypercapnia exposed red drum (Sciaenops ocellatus). *Journal of Comparative Physiology B* **186**, 447–460 (2016).
76. Wood, C. M. & Eom, J. The internal CO2 threat to fish: high PCO2 in the digestive tract. *Proceedings of the Royal Society B: Biological Sciences* **286**, 20190832 (2019).
77. Gam, L. T. H. *et al.* Effects of temperature on acid–base regulation, gill ventilation and air breathing in the clown knifefish, Chitala ornata. *Journal of Experimental Biology* **223**, jeb216481 (2020).
78. Kwan, G. T. & Tresguerres, M. Elucidating the acid-base mechanisms underlying otolith overgrowth in fish exposed to ocean acidification. *Science of The Total Environment* **823**, 153690 (2022).
79. Montgomery, D. W. *et al.* Rapid blood acid–base regulation by European sea bass (Dicentrarchus labrax) in response to sudden exposure to high environmental CO2. *Journal of Experimental Biology* **225**, jeb242735 (2022).
80. Perry, S. F. & Gilmour, K. M. Sensing and transfer of respiratory gases at the fish gill. *Journal of Experimental Zoology* **293**, 249–263 (2002).
81. Harter, T. S. *et al.* A solution to Nature's haemoglobin knockout: a plasma-accessible carbonic anhydrase catalyses CO2 excretion in Antarctic icefish gills. *Journal of Experimental Biology* **221**, jeb190918 (2018).
82. Chung, M.-T., Trueman, C. N., Godiksen, J. A. & Grønkjær, P. Otolith δ13C values as a metabolic proxy: approaches and mechanical underpinnings. *Mar Freshw Res* **70**, 1747–1756 (2019).
83. Weber, J. N. & Woodhead, P. M. J. Carbon and oxygen isotope fractionation in the skeletal carbonate of reef-building corals. *Chem Geol* **6**, 93–117 (1970).
84. Helser, T., McKay, J. & Kastelle, C. *Stable Isotope Age Validation of Pacific Cod (NPRB Report 1105)*. https://nprb.org/project-search/#metadata/78f51e6c-9ba6-4425-86d2-e91fbf1818a8/project/files (2014).

85. Kastelle, C. R. *et al.* Age validation of Pacific cod (Gadus macrocephalus) using high-resolution stable oxygen isotope (δ 18O) chronologies in otoliths. *Fish Res* **185**, 43–53 (2017).
86. Roberson, N. RACEBASE Database of fisheries surveys. . *RACEBASE Database* 1953–2022 Preprint at (2022).
87. Beverton, R. J. H. & Holt, S. J. *On the Dynamics of Exploited Fish Populations*. (Springer, Dordrecht, 1957). doi:10.1007/978-94-011-2106-4.
88. Laurel, B. J., Hurst, T. P., Copeman, L. A. & Davis, M. W. The role of temperature on the growth and survival of early and late hatching Pacific cod larvae (Gadus macrocephalus). *J Plankton Res* **30**, 1051–1060 (2008).
89. Marsh, J. M., Hillgruber, N. & Foy, R. J. Temporal and ontogenetic variability in trophic role of four groundfish Species—walleye pollock, Pacific cod, arrowtooth flounder, and Pacific halibut—around Kodiak Island in the Gulf of Alaska. *Trans Am Fish Soc* **141**, 468–486 (2012).
90. Schell, D. M., Barnett, B. A. & Vinette, K. A. Carbon and nitrogen isotope ratios in zooplankton of the Bering, Chukchi and Beaufort seas. *Mar Ecol Prog Ser* **162**, 11–23 (1998).
91. Kline, T. C. Jr. Temporal and spatial variability of 13C/12C and 15N/14N in pelagic biota of Prince William Sound, Alaska. *Canadian Journal of Fisheries and Aquatic Sciences* **56**, 94–117 (1999).
92. Max, L. *et al.* Pulses of enhanced North Pacific Intermediate Water ventilation from the Okhotsk Sea and Bering Sea during the last deglaciation. *Climate of the Past* **10**, 591–605 (2014).
93. Bauch, D., Polyak, L. & Ortiz, J. D. A baseline for the vertical distribution of the stable carbon isotopes of dissolved inorganic carbon (δ13CDIC) in the Arctic Ocean. *arktos* **1**, 15 (2015).
94. Niebauer, H. J. Sea ice and temperature variability in the eastern Bering Sea and the relation to atmospheric fluctuations. *J Geophys Res Oceans* **85**, 7507–7515 (1980).
95. Wang, J., Hu, H., Mizobata, K. & Saitoh, S. Seasonal variations of sea ice and ocean circulation in the Bering Sea: A model-data fusion study. *J Geophys Res Oceans* **114**, (2009).
96. Cross, J., Monacci, N., Musielewicz, S. & Maenner Jones, S. High-resolution ocean and atmosphere pCO2 time-series measurements from mooring M2 164W 57N in the Bering Sea (NCEI Accession 0157599). 2013–2019 Preprint at https://doi.org/10.3334/cdiac/otg.tsm_m2_164w_57n (2019).
97. Nichol, D. G., Kotwicki, S. & Zimmermann, M. Diel vertical migration of adult Pacific cod Gadus macrocephalus in Alaska. *J Fish Biol* **83**, 170–189 (2013).
98. Pilcher, D. J. *et al.* Dynamically downscaled projections of ocean acidification for the Bering Sea. *Deep Sea Research Part II: Topical Studies in Oceanography* **198**, 105055 (2022).
99. Pilcher, D. J. *et al.* Modeled effect of coastal biogeochemical processes, climate variability, and ocean acidification on aragonite saturation state in the Bering Sea. *Front Mar Sci* **5**, (2019).
100. Takahashi, T., Sutherland, S. C., Feely, R. A. & Wanninkhof, R. Decadal change of the surface water pCO2 in the North Pacific: A synthesis of 35 years of observations. *J Geophys Res Oceans* **111**, (2006).

101. Hurst, T. P. *et al.* Effects of ontogeny, temperature, and light on vertical movements of larval Pacific cod (Gadus macrocephalus). *Fish Oceanogr* **18**, 301–311 (2009).
102. Laurel, B. J., Ryer, C. H., Knoth, B. & Stoner, A. W. Temporal and ontogenetic shifts in habitat use of juvenile Pacific cod (Gadus macrocephalus). *J Exp Mar Biol Ecol* **377**, 28–35 (2009).
103. Helser, T. *et al.* A 200-year archaeozoological record of Pacific cod (Gadus macrocephalus) life history as revealed through ion microprobe oxygen isotope ratios in otoliths. *J Archaeol Sci Rep* **21**, 1236–1246 (2018).
104. Froese, R. & Pauly, D. FishBase fish species database. Preprint at https://www.fishbase.se/search.php (2022).
105. Rau, G. H., Sweeney, R. E. & Kaplan, I. R. Plankton 13C: 12C ratio changes with latitude: differences between northern and southern oceans. *Deep Sea Research Part A. Oceanographic Research Papers* **29**, 1035–1039 (1982).
106. Popp, B. N. *et al.* Effect of Phytoplankton Cell Geometry on Carbon Isotopic Fractionation. *Geochim Cosmochim Acta* **62**, 69–77 (1998).
107. Boyer, T. *et al.* World Ocean Atlas 2018 database. *World Ocean Atlas* 1953–2018 Preprint at https://www.ncei.noaa.gov/archive/accession/NCEI-WOA18 (2018).
108. Lauvset, S. K. *et al.* A new global interior ocean mapped climatology: the 1° × 1° GLODAP version 2. *Earth Syst Sci Data* **8**, 325–340 (2016).
109. Lauvset, S. K. *et al.* GLODAPv2.2022: the latest version of the global interior ocean biogeochemical data product. *Earth Syst Sci Data* **14**, 5543–5572 (2022).
110. Lauvset, S. K. *et al.* The annual update GLODAPv2.2023: the global interior ocean biogeochemical data product. *Earth Syst Sci Data* **16**, 2047–2072 (2024).
111. Eide, M., Olsen, A., Ninnemann, U. S. & Johannessen, T. A global ocean climatology of preindustrial and modern ocean δ13C. *Global Biogeochem Cycles* **31**, 515–534 (2017).
112. Kalish, J. M. Oxygen and carbon stable isotopes in the otoliths of wild and laboratory-reared Australian salmon (Arripis trutta). *Mar Biol* **110**, 37–47 (1991).
113. Thorrold, S. R., Jones, C. M. & Campana, S. E. Response of otolith microchemistry to environmental variations experienced by larval and juvenile Atlantic croaker (Micropogonias undulatus). *Limnol Oceanogr* **42**, 102–111 (1997).
114. Edmonds, J. S., Steckis, R. A., Moran, M. J., Caputi, N. & Morita, M. Stock delineation of pink snapper and tailor from Western Australia by analysis of stable isotope and strontium/calcium ratios in otolith carbonate. *J Fish Biol* **55**, 243–259 (1999).
115. Stephenson, P. C., Edmons, J. S., Moran, M. J. & Caputi, N. Analysis of stable isotope ratios to investigate stock structure of red emperor and Rankin cod in northern Western Australia. *J Fish Biol* **58**, 126–144 (2001).
116. Ashford, J. & Jones, C. Oxygen and carbon stable isotopes in otoliths record spatial isolation of Patagonian toothfish (Dissostichus eleginoides). *Geochim Cosmochim Acta* **71**, 87–94 (2007).
117. Nelson, J., Hanson, C., Koenig, C. & Chanton, J. Influence of diet on stable carbon isotope composition in otoliths of juvenile red drum Sciaenops ocellatus. *Aquat Biol* **13**, 89–95 (2011).
118. Wells, R. J. D., Rooker, J. R. & Prince, E. D. Regional variation in the otolith chemistry of blue marlin (Makaira nigricans) and white marlin (Tetrapturus albidus) from the western North Atlantic Ocean. *Fish Res* **106**, 430–435 (2010).

119. Correia, A. T., Barros, F. & Sial, A. N. Stock discrimination of European conger eel (Conger conger L.) using otolith stable isotope ratios. *Fish Res* **108**, 88–94 (2011).
120. Trueman, C. N., Rickaby, R. E. M. & Shephard, S. Thermal, trophic and metabolic life histories of inaccessible fishes revealed from stable-isotope analyses: a case study using orange roughy *Hoplostethus atlanticus*. *J Fish Biol* **83**, 1613–1636 (2013).
121. Howard, E. M. *et al.* Climate-driven aerobic habitat loss in the California Current System. *Sci Adv* **6**, (2020).
122. Penn, J. L. & Deutsch, C. Geographical and taxonomic patterns in aerobic traits of marine ectotherms. *Philosophical Transactions of the Royal Society B: Biological Sciences* **379**, (2024).
123. Logan, J. M. & Lutcavage, M. E. Assessment of trophic dynamics of cephalopods and large pelagic fishes in the central North Atlantic Ocean using stable isotope analysis. *Deep Sea Research Part II: Topical Studies in Oceanography* **95**, 63–73 (2013).
124. Sherwood, G. & Rose, G. Influence of swimming form on otolith δ13C in marine fish. *Mar Ecol Prog Ser* **258**, 283–289 (2003).
